# Calculating the Effects of Longitudinal Resistance in Multi-Series-Connected Quantum Hall Effect Devices

**DOI:** 10.6028/jres.103.037

**Published:** 1998-12-01

**Authors:** M. E. Cage, A. Jeffery, R. E. Elmquist, K. C. Lee

**Affiliations:** National Institute of Standards and Technology, Gaithersburg, MD 20899-0001

**Keywords:** ac quantum Hall effect, equivalent electrical circuit, longitudinal resistance, multi-series connections, quantized Hall resistance

## Abstract

Many ac quantized Hall resistance experiments have measured significant values of ac longitudinal resistances under temperature and magnetic field conditions in which the dc longitudinal resistance values were negligible. We investigate the effect of non-vanishing ac longitudinal resistances on measurements of the quantized Hall resistances by analyzing equivalent circuits of quantized Hall effect resistors. These circuits are based on ones reported previously for dc quantized Hall resistors, but use additional resistors to represent longitudinal resistances. For simplification, no capacitances or inductances are included in the circuits. The analysis is performed for many combinations of multi-series connections to quantum Hall effect devices. The exact algebraic solutions for the quantized Hall resistances under these conditions of finite ac longitudinal resistances provide corrections to the measured quantized Hall resistances, but these corrections do not account for the frequency dependences of the ac quantized Hall resistances reported in the literature.

## 1. Introduction

In the integer quantum Hall effect (QHE) [1–3], the Hall resistance *R*_H_ of the *i*th plateau of a fully-quantized, two-dimensional electron gas (2DEG) is *R*_H_(*i*) = *V*_H_(*i*)/*I*_T_, where *V*_H_(*i*) is the quantum Hall voltage measured between potential probes located on opposite sides of the device, and *I*_T_ is the total current flowing between the source and drain current contacts at the ends of the device. Under ideal conditions, the values of *R*_H_(*i*) in standards-quality devices satisfy the relationships *R*_H_(*i*) = *h*/(*e*^2^*i*) = *R*_K_/*i*, where *h* is the Planck constant, *e* is the elementary charge, *i* is an integer, and *R*_K_ is the von Klitzing constant *R*_K_ ≈ 25 812.807 Ω. However, the conditions are not always ideal. We will assume that the values of *R*_H_(*i*) can vary with the device temperature *T* and with the frequency *f* of the applied ac current (although the equations will not explicitly indicate *T* or *f*). Thus the measured values of *R*_H_(*i*) will usually not be equal to *h*/(*e*^2^*i*) in this paper.

In the dc quantum Hall effect the current flow within the 2DEG is nearly dissipationless within the quantum Hall plateau regions of high-quality devices. The longitudinal resistance *R_x_*(*i*) = *V_x_*(*i*)/*I*_T_, where *V_x_*(*i*) is the longitudinal voltage drop between potential probes located on the same side of the device, becomes very small over ranges of magnetic field over which *R*_H_(*i*) exhibits plateaus. The values of *R_x_*(*i*) increase with increasing temperature.

Many laboratories are now attempting to employ the QHE to realize an intrinsic ac resistance standard by using an ac ratio bridge to compare the ac quantized Hall resistance *R*_H_ with ac reference standards. Measured values [4–9] of the ac quantized Hall resistance *R*_H_ are reported to vary with the frequency of the applied current (usually increasing linearly with frequency), and differ from the dc value by more than 10^−7^ *R*_H_(*i*) at a frequency of 1592 Hz (angular frequency *ω* = 2*πf* = 10^4^ rad/s). With one notable exception [10], the reported ac longitudinal resistances are significantly larger than the dc longitudinal resistances in the same device under the same temperature and magnetic field conditions. The ac longitudinal resistances increase with increasing frequency of the applied current, and are of order 1 mΩ at 1592 Hz [4, 5, 11]. The frequency dependences of *R*_H_ and *R*_x_ are reported to be in the real, resistive (in-phase) component of the ac impedance measurements.

These effects might be caused by intrinsic properties of the quantum Hall devices. However, calculations [12] of the intrinsic impedance due to the Hall capacitance of the two-dimensional electron gas across the quantum Hall device have provided no plausible intrinsic impedance explanations for the observed frequency dependences of the ac quantized Hall resistance *R*_H_. Furthermore, neither the kinetic inductance [12] nor the magnetic inductance [12] of the device can explain the observed frequency dependences of the ac longitudinal resistance *R_x_*. Even if the intrinsic impedances considered in [12] were significant, they would primarily affect the imaginary (reactive) component of the impedance not the real (resistive) component *R*_H_.

The observed frequency dependences of *R*_H_ and *R_x_* could arise from problems in the measurement systems, from the large impedances in the sample probes, or from uncorrected frequency dependences in the ac reference standards. These possible problems should all be addressed, but in this paper we assume that there are indeed significant in-phase ac longitudinal impedances (the resistances along the devices) as reported, and investigate what effect real longitudinal resistances would have on the measured in-phase ac values of *R*_H_.

We use equivalent electrical circuits of a QHE device to investigate the effects of these non-vanishing longitudinal resistances on the quantized Hall resistance measurements. The analysis soon becomes non-trivial. To simplify the analysis, only in-phase components are considered in the circuits. We ignore all capacitances and inductances. The effects of electrical shielding and leakage resistances are also not included. All multi-series connections [13] to the devices used in the literature are considered. The algebraic equations are exact and sometimes lengthy. It is important that the solutions be exact because we are looking for explanations of very small, but significant, experimental effects. The final equations are presented to alleviate the need for others to perform the task of deriving them. All the equations have been independently derived by at least two of the authors, and then numerically confirmed with computer software.

## 2. Equivalent Circuits

Two different equivalent electrical circuits for standards-quality QHE devices operated under ideal dc conditions when *R*_H_(*i*) = *h*/(*e*^2^*i*) = *R*_K_/*i* have been described in the literature: the “diamond-array” circuit of Ricketts and Kemeny [14] and the “ring-array” circuit of Delahaye [13]. The algebraic equations of both circuits are identical in the absence of longitudinal resistance. We use the circuit of Ricketts and Kemeny [14] with little alteration, except that *R*_H_(*i*) is allowed to vary with temperature and frequency, and can thereby differ from *h*/(*e*^2^*i*); and we include longitudinal resistances at the source and the drain ends of the device. The circuit of Delahaye [13], however, was derived using the assumption that the longitudinal resistance vanished. In order to account for non-zero longitudinal resistance, we add resistors at appropriate places in the circuit, and again allow *R*_H_(*i*) to be a function of temperature and frequency.

We show that when longitudinal resistance is included, the results calculated using the two circuits are similar, but the algebraic solutions are much simpler with the diamond-array circuit. The diamond-array circuit will therefore be used for all multi-series connection calculations. However, we present solutions to the simplest multi-series ring-array in Appendix A to demonstrate the added complexity of analyzing with that circuit.

### 2.1 Diamond-Array Circuit

**Fig. 1:**
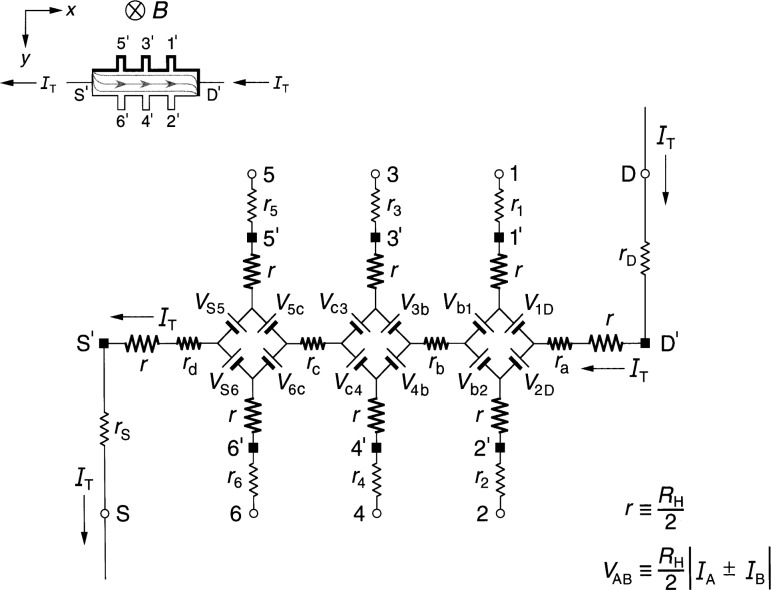
Equivalent circuit, using diamond-arrays of internal voltage generators *V*_AB_, of a quantum Hall effect device when the device is operated on a quantized Hall resistance plateau and has longitudinal resistance. The symbols and figure inset are explained in Sec. 2.1.

Figure 1 shows the QHE device equivalent circuit of Ricketts and Kemeny [14] for the case when (a) the magnetic flux density *B* shown in the inset is directed into the figure; and (b) at an instant when a positively-charged applied current *I*_T_ enters the device drain contact pad D*′* and exits the source contact pad S*′*. Under these conditions the drain contact pad D′ and the potential probe contact pads 1*′*, 3*′*, and 5*′* at the device periphery are at higher potentials than contact pads S*′*, 2*′*, 4*′*, and 6*′*. The higher potentials are represented in the inset by thicker lines on the device periphery. The curves within the device show the current flow pattern for this case. The arrows pointing in the opposite direction to *I*_T_ indicate the direction of motion of electrons, and are reminders to the reader that the current within the device is composed of electrons passing through the 2DEG, rather than positive charges. The *x* axis is directed along the device, with the *y* axis across the device.

Every arm of the equivalent circuit has an intrinsic resistance *r* whose value is

$$r\equiv \frac{R_{H}\left(i\right)}{2}=\frac{V_{H}\left(i\right)}{2I_{T}}.$$ (1)

Note that *R*_H_(*i*) can be a function of temperature and frequency, and can differ from the value *h*/(*e*^2^*i*). There is electrical access to the device at connection points S, 1 through 6, and D. Each external arm of the circuit has a lead resistance *r*_S_, *r*_1_ through *r*_6_, or *r*_D_ which includes the contact resistance to the 2DEG, the wire resistance connecting a contact pad on the device to a sample probe lead, and the inner conductor resistance of that coaxial sample probe lead. The lead resistance values vary with the liquid helium level in the sample probe. They can be measured pair-wise as a function of liquid helium level via two-terminal resistance measurements when the quantum Hall device is replaced by electrical shorts at positions S*′*, 1*′* through 6*′*, and D*′*. The lead resistances are typically each about 1 Ω in ac quantized Hall resistance experiments.

The potentials at the ends of the arms at points S, 1 through 6, and D are produced by diamond-shaped arrays of voltage generators, where *V*_AB_ is the generator located between arms A and B, and produces a voltage defined by

$$V_{\text{AB}}\equiv \frac{R_{H}}{2}\left|I_{A}\pm I_{B}\right|,$$ (2)

where *I_A_* and *I*_B_ are the magnitudes of the current flowing in arms A and B of the circuit. The currents *I_A_* and *I*_B_ within the absolute quantity sign of Eq. (2) are added if they both enter or both leave the voltage generator, and are subtracted if one current enters and the other current leaves the generator. Since the voltages produced by the voltage generators are functions of *R*_H_(*i*), their values can vary with temperature and frequency. The arms A and B in the diamond-array voltage generator definitions can be the external arms S, 1 through 6, and D *or* the two internal segments containing resistances *r*_b_ and *r*_c_. Hence all of the voltage generators in Fig. 1 have magnitudes *V*_AB_ = [*R*_H_(*i*)/2]*I*_T_ because there are not currents in potential arms 1 through 6. For clarity, the voltage generators are indicated in the figure as batteries whose positive terminals are oriented to give the correct potentials at the end of each arm. The applied ac current *I*_T_ alternates direction, so the voltage generators reverse sign each half cycle. Thus, for the part of the period in which *I*_T_ flows in the direction indicated in Fig. 1, the voltage generators have the polarities shown. Half a period later *I*_T_ changes direction, and all the voltage generators reverse polarities.

The circuit elements *r*_a_, *r*_b_, *r*_c_, and *r*_d_ in Fig. 1 represent real (in-phase) longitudinal resistances within the device. These resistances are functions of temperature and frequency. Longitudinal resistances are obtained by potential difference measurements along a side of the device in the *x* direction. For example, the longitudinal resistance *R_x_*(2, 6) between points 2 and 6 is

$$R_{x}\left(2,6\right)\equiv \frac{V_{x}\left(2,6\right)}{I_{T}}=\frac{\left[V_{2}-V_{6}\right]}{I_{T}},$$ (3)

where *V_x_*(2,6) is the voltage difference measured between points 2 and 6. *V*_2_ is the potential at point 2 relative to the circuit ground (which would be located at point S when making four-terminal resistance measurements). *V*_6_ is the potential at point 6, and *I*_T_ = *V*_R_/*R*_R_ is determined by measuring the voltage drop *V*_R_ across a reference resistance *R*_R_ connected in series with the quantum Hall device. Then, according to Fig. 1, *V**_x_*(2,6) = *V*_6c_ + *r*_c_*I*_T_ – *V*_c4_ + *V*_4b_ + *r*_b_*I*_T_ – *V*_b2_. Since no current flows through leads 2, 4, or 6 in Fig. 1, *V*_6c_ = *V*_c4_ = *V*_4b_ = *V*_b2_ = [*R*_H_(*i*)/2]*I*_T_. Therefore

$$R_{x}\left(2,6\right)=R_{x}\left(1,5\right)=r_{b}+r_{c}.$$ (4)

Sample probes used in dc QHE measurements have a pair of leads to the source contact pad S*′* and another pair to the drain contact pad D*′*. Only one lead of each pair carries the current *I*_T_, so all four dc resistances *r*_a_, *r*_b_, *r*_c_, and *r*_d_ can be measured. Sample probes for the ac QHE, however, have a single coaxial lead to each of the contact pads in order to reduce heat loss. Therefore only *r*_b_ and *r*_c_ can be determined directly via ac measurements of *V_x_*(4,2) and *V_x_*(6,4). Values for *r*_a_ and *r*_d_ could be estimated from their dc *r*_a_/*r*_b_ and *r*_d_/*r*_c_ ratios if the *r*_b_/*r*_c_ ratio is the same for both ac and dc measurements. Typical ac *r*_b_ and *r*_c_ values are reported [4,5,11] to be about 1 mΩ at 1592 Hz.

The quantized Hall resistance *R*_H_(3,4) measured between points 3 and 4 in Fig. 1 is

$$R_{H}\left(3,4\right)\equiv \frac{V_{H}(3,4)}{I_{T}}=\frac{\left[V_{3}-V_{4}\right]}{I_{T}}=\frac{\left[V_{c4}+V_{c3}\right]}{I_{T}}=R_{H}(i).$$ (5)

The device shown in Fig. 1 is homogeneous, i.e., the quantized Hall resistances *R*_H_ are *all* measured on plateau regions, their values are the *same* between *all* the Hall potential probe sets, and they are all measured at the *same* magnetic flux density. Therefore

$$R_{H}\left(1,2\right)=R_{H}\left(3,4\right)=R_{H}\left(5,6\right)=R_{H}\left(i\right).$$ (6)

Note once again that *R*_H_(*i*) can be a function of temperature and frequency, and can differ from the ideal value *h*/(*e*^2^*i*). This equivalent circuit of Ricketts and Kemeny [14] satisfies the conditions of a quantum Hall effect device.

### 2.2 Ring-Array Circuit

**Fig. 2:**
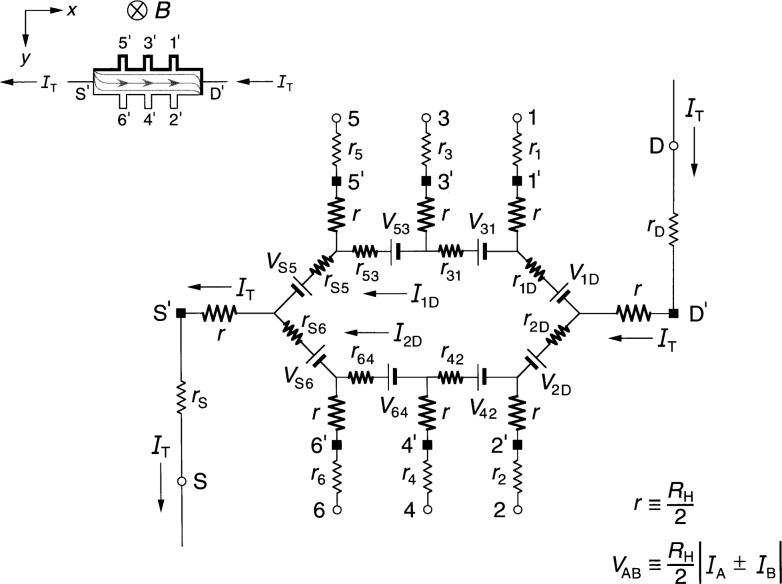
Equivalent circuit, using ring-arrays of internal voltage generators *V*_AB_, of a quantum Hall effect device when the device is operated on a quantized Hall resistance plateau and has longitudinal resistance. The symbols are explained in Sec. 2.2.

Figure 2 shows a generalized form of the ring-array equivalent circuit of Delahaye [13] for the same current and magnetic flux density directions as Fig. 1. Resistances *r*_1D_, *r*_2D_, through *r*_S6_ have been placed in series with the voltage generators to simulate real (in-phase) longitudinal resistances within the device. These series resistances could be thought of as internal resistances of the voltage generators. Once again, every arm of the circuit has a lead resistance *r*_S_, *r*_1_ through *r*_6_, and *r*_D_, and an intrinsic resistance *r* whose value is *R*_H_(*i*)/2. The values of *R*_H_(*i*) can be functions of temperature and frequency, and are not necessarily equal to *h*/(*e*^2^*i*). The resistances *r*_1D_, *r*_2D_, through *r*_S6_ can also be functions of temperature and frequency.

The potentials at the ends of the arms at points S, 1 through 6, and D are now produced by a ring-shaped array of voltage generators, where *V*_AB_ denotes the voltage produced by the generator located between external arms A and B, and is again defined by *V*_AB_ ≡ (*R*_H_/2)|*I*_A_ ± *I*_B_|. The currents are added if *I*_A_ and *I*_B_ both enter or both leave the voltage generator, and are subtracted if one current enters and the other current leaves the generator. It is important to note that the arms A and B in the ring-array voltage generator definitions include only currents in the *external* arms S, 1 through 6, and D, *not* in the eight *internal* segments containing resistances *r*_1D_ through *r*_S6_. Because there are no currents in arms 1 through 6, the generators *V*_31_, *V*_42_, *V*_53_, and *V*_64_ produce zero voltage in Fig. 2. The voltage generators *V*_1D_, *V*_2D_, *V*_S5_, and *V*_S6_ have magnitudes *V*_AB_ = [*R*_H_(*i*)/2]*I*_T_.

We again measure potential differences *V*(A,B) around the periphery of the device between points S, 1 through 6, and D, and then assign values to the voltage generators *V*_AB_ and series resistances *r*_AB_ of the equivalent circuit. Unique assignments can not be made because there are nine unknown resistances (*r* and the eight values of *r*_AB_) and eight unknown voltage generators, but only seven independent voltages which can be measured (three quantum Hall voltages and four longitudinal voltages). Therefore, we find the simplest solution that is consistent with the results of Sec. 2.1, and assume in Fig. 2 that (a) the sum of resistances in both the top half and the bottom half of the ring are equal, i.e., that *r*_1D_ + *r*_31_ + *r*_53_ *+ r*_S5_ = *r*_2D_ + *r*_42_ + *r*_64_ + *r*_S6_, and (b) that the device is homogeneous. Therefore the currents in the top half and the bottom half of the ring, *I*_1D_ and *I*_2D_, are both equal to *I*_T_/2. This means that

$$R_{x}(2,6)=R_{x}(1,5)=\frac{r_{42}+r_{64}}{2}=\frac{r_{31}+r_{53}}{2}$$ (7)

if *R_x_*(2,6) is still defined to be *R_x_*(2,6) ≡ *V_x_*(2,6)/*I*_T_. Equation (7) still does not uniquely define the resistances *r*_42_, *r*_64_, *r*_31_, and *r*_53_, but we can assure that Eq. (7) is identical to Eq. (4) by letting *r*_42_ = *r*_31_ = 2*r*_b_ and *r*_64_ = *r*_53_ = 2*r*_c_, where *r*_b_ and *r*_c_ are defined in Fig. 1. The ring-array circuit of voltage generators distributes the current within the device into two paths, which is the reason for the factors of two in the longitudinal resistances. It follows that

$$R_{H}\left(1,2\right)=R_{H}\left(3,4\right)=R_{H}\left(5,6\right)=R_{H}\left(i\right)$$ (8)

only if *r*_S6_ = *r*_S5_ and *r*_2D_ = *r*_1D_. This also requires that *r*_2D_ = *r*_1D_ = 2*r*_a_ and *r*_S6_ = *r*_S5_ = 2*r*_d_ for Eq. (8) to be consistent with Eq. (5). We emphasize that the assumptions *r*_2D_ = *r*_1D_ = 2*r*_a_, *r*_42_ = *r*_31_ = 2*r*_b_, *r*_64_ = *r*_53_ = 2*r*_c_, and *r*_S6_ = *r*_S5_ = 2*r*_d_ were made so that the solution for the circuit in Fig. 2 is consistent with that of Fig. 1.

We have also derived the equations for *R*_H_ and *R_x_* using the equivalent circuit of Fig. 2 without making the simplifying assumptions described above, but have not included those equations because they are very complicated. The simplest solution described above is adequate to model this circuit.

In addition, we have derived the current and voltage equations for the equivalent circuit of Fig. 2 using Delahaye’s [4] assumption that the resistances labeled *r* have the ideal value *h*/(*e*^2^*i*), and that the voltage generators produce ideal voltages *V*_AB_ = *h*/(2*e*^2^*i*)|*I*_A_ ± *I*_B_|. The values of resistances *r*_1D_, *r*_2D_, through *r*_S6_ are adjusted to give the measured values of *R*_H_(*i*) and *R_x_* around the device periphery. The equations are very complicated, but numerical results using typical experimental values for the circuit elements are equivalent. For this reason, we use the method of adjusting the value of *R*_H_(*i*) to agree with quantized Hall resistance measurements because that method yields much simpler equations and identical numerical results.

## 3. Non-Uniform Quantum Hall Voltage Distributions

We assumed in Fig. 1 and Fig. 2 that the quantized Hall voltages *V*_H_ are all measured on plateau regions where *V*_H_ is independent of the magnetic flux density, and that the resulting quantized Hall resistances *R*_H_ are the same for all the Hall potential probe sets at the same magnetic flux density. These conditions will certainly be necessary when making multiple-series connections to the same device. But what if one of the Hall probe potential sets has a value of the quantized Hall resistance $R_{H}^{\prime }$ that differs from the *R*_H_ value of the other two sets? This section examines that particular inhomogeniety, which can occur if the device (a) has a nonuniform 2DEG density due to improper fabrication techniques; (b) suffers environmental deterioration of the surface; (c) is operated at temperatures that are too high [15]; (d) is cooled too rapidly; or (e) has one or more contacts with nonnegligible contact resistance.

### 3.1 Diamond-Array Circuit Results

**Fig. 3:**
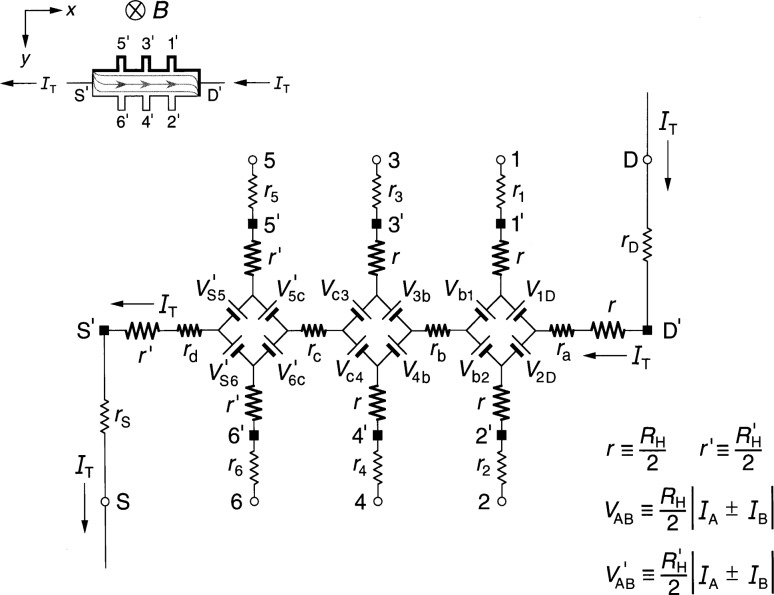
Equivalent circuit using diamond-arrays of voltage generators when the left end of the device has a quantized Hall resistance value $R_{H}^{\prime }$ and the rest of the device remains at *R*_H_(*i*). The results are given in Sec. 3.1.

Figure 3 shows the diamond-array-based equivalent circuit when the left end of the device has a quantized Hall resistance value $R_{H}^{\prime }$ (*i*) between probes 5 and 6 and the remainder of the device is still at *R*_H_(*i*). In this case the Hall voltages are

$$V_{H}\left(1,2\right)=V_{H}\left(3,4\right)=R_{H}\left(i\right)I_{T}$$ (9a)

$$V_{H}\left(5,6\right)\equiv R_{H}\left(5,6\right)I_{T}=R_{H}^{\prime }\left(i\right)I_{T}$$ (9b)

and the longitudinal voltages are

$$V_{x}\left(4,6\right)\equiv R_{x}\left(4,6\right)I_{T}=\left\{r_{c}+\left[\frac{R_{H}^{\prime }-R_{H}}{2}\right]\right\}I_{T}$$ (10a)

$$V_{x}\left(3,5\right)\equiv R_{x}\left(3,5\right)I_{T}=\left\{r_{c}-\left[\frac{R_{H}^{\prime }-R_{H}}{2}\right]\right\}I_{T}.$$ (10b)

The longitudinal resistance *r*_c_ along this section of the device is therefore the average of *R_x_*(4, 6) and *R_x_*(3, 5). The longitudinal voltages *V_x_*(2, 4) ≡ *R_x_*(2, 4)*I*_T_ = *r*_b_*I*_T_ and *V_x_*(1, 3) ≡ *R_x_*(1, 3)*I*_T_ = *r*_b_*I*_T_ remain the same as in Sec. 2.1.

### 3.2 Ring-Array Circuit Results

**Fig. 4:**
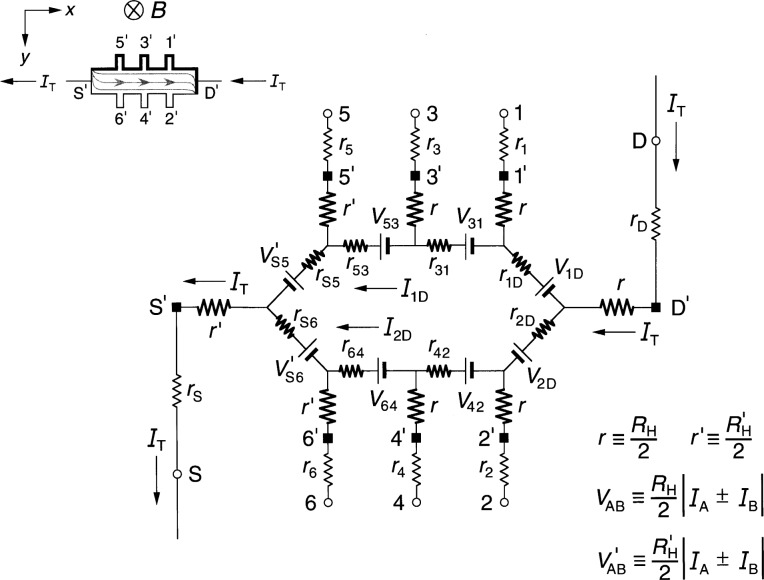
Equivalent circuit using ring-arrays of voltage generators when the left end of the device has a quantized Hall resistance value $R_{H}^{\prime }$(*i*) and the rest of the device remains at *R*_H_(*i*). The results are given in Sec. 3.2.

Figure 4 shows the ring-array-based equivalent circuit when the left end of the device has a quantized Hall resistance $R_{H}^{\prime }$(*i*) and the rest of the device is still at *R*_H_(*i*). We label the internal currents in the top and bottom halves of the ring as *I*_1D_ and *I*_2D_, respectively. Note that even though the top and bottom halves of the circuit appear to be symmetrical, *I*_1D_ does not have to be equal to *I*_2D_, and in fact is not equal, as will be shown. In order for the quantum Hall voltage to be

$$V_{H}\left(5,6\right)=R_{H}\left(5,6\right)I_{T}=R_{H}^{\prime }\left(i\right)I_{T}$$ (11a)

it is necessary that

$$r_{\text{S}5}I_{1D}=r_{\text{S}6}I_{2D},$$ (11b)

and in order for

$$V_{H}\left(3,4\right)\equiv R_{H}\left(3,4\right)I_{T}=R_{H}\left(i\right)I_{T},$$ (12a)

the internal current in the bottom leg of the circuit must be

$$I_{2D}=I_{T}-I_{1D}=\frac{\left[(R_{H}^{\prime }-R_{H})+r_{53}\right]}{\left[r_{53}+r_{64}\right]}\;I_{T}.$$ (12b)

Furthermore, in order that

$$V_{x}\left(4,6\right)\equiv R_{x}(4,6)I_{T}=\left\{r_{c}+\left[\frac{R_{H}^{\prime }-R_{H}}{2}\right]\right\}I_{T}$$ (13a)

and

$$V_{x}\left(3,5\right)\equiv R_{x}\left(3,5\right)I_{T}=\left\{r_{c}-\left[\frac{R_{H}^{\prime }-R_{H}}{2}\right]\right\}I_{T},$$ (13b)

as in the diamond-array case, *r*_64_ = *r*_53_ = 2 *r*_c_, which is the same assumption made in Sec. 2.2 for the case of the homogeneous device. However, we find below that *r*_S6_ ≠ *r*_S5_ ≠2*r*_d_, *r*_31_ ≠*r*_42_ ≠2*r*_b_, and *r*_1D_ ≠*r*_2D_ ≠2*r*_a_.

The combination of Eq. (11b) and Eq. (12b) yields the result

$$\frac{r_{S6}}{r_{S5}}=\frac{I_{1D}}{I_{2D}}=\frac{\left[2r_{c}-(R_{H}^{\prime }-R_{H})\right]}{\left[2r_{c}+(R_{H}^{\prime }-R_{H})\right]},$$ (13c)

so *r*_S6_ ≠ *r*_S5_ ≠ 2*r*_d_ and *I*_1D_ ≠ *I*_2D_.

We also require

$$V_{H}\left(1,2\right)\equiv R_{H}\left(1,2\right)I_{T}=R_{H}\left(i\right)I_{T},$$ (14a)

so

$$\frac{r_{42}}{r_{31}}=\frac{\left[2r_{c}-(R_{H}^{\prime }-R_{H})\right]}{\left[2r_{c}+(R_{H}^{\prime }-R_{H})\right]}=\frac{I_{1D}}{I_{2D}}$$ (14b)

and

$$\frac{r_{2D}}{r_{1D}}=\frac{I_{1D}}{I_{2D}}.$$ (14c)

Since the voltages must sum to zero around the ring of voltage generators and internal resistances, *I*_2D_ must also satisfy the condition

$$I_{2D}=\frac{\left[(R_{H}^{\prime }-R_{H})+(r_{1D}+r_{31}+r_{53}+r_{\text{S}5})\right]}{\left[(r_{1D}+r_{31}+r_{53}+r_{\text{S}5})+(r_{2D}+r_{42}+r_{64}+r_{\text{S}6}\right)}\;I_{T},$$ (14d)

as well as Eq. (12b).

Because of the larger number of unknown variables, the solutions derived using the ring-array circuit are more complicated than those for the diamond-shaped array of Sec. 3.1. Furthermore, many of the internal resistances are expressed as ratios relative to other resistances in the circuit, rather than unique values. However, they could be assigned unique values by letting *r*_64_ = *r*_53_ = 2*r*_c_, *r*_S6_ = *r*_42_ = *r*_2D_ = [2*r*_c_ – ( $R_{H}^{\prime }$ – *R*_H_)], and *r*_S5_ = *r*_31_ = *r*_1D_ = [2*r*_c_ + ( $R_{H}^{\prime }$ – *R*_H_)].

## 4. Load Resistance Across the Device

Ricketts and Kemeny [14] tested their equivalent circuit experimentally by placing a load resistor across one pair of Hall potential probes. We consider the effects of a load resistor in this section for both diamond-array and ring-array circuits. The results may explain the observations of negative values of *V_x_* reported by Ricketts and Kemeny [14]. The equations for *V*_H_ and *V_x_* are not the same for the two equivalent circuits, but the numerical results are nearly identical if representative experimental values are used for the circuit elements.

### 4.1 Diamond-Array Circuit Results

**Fig. 5:**
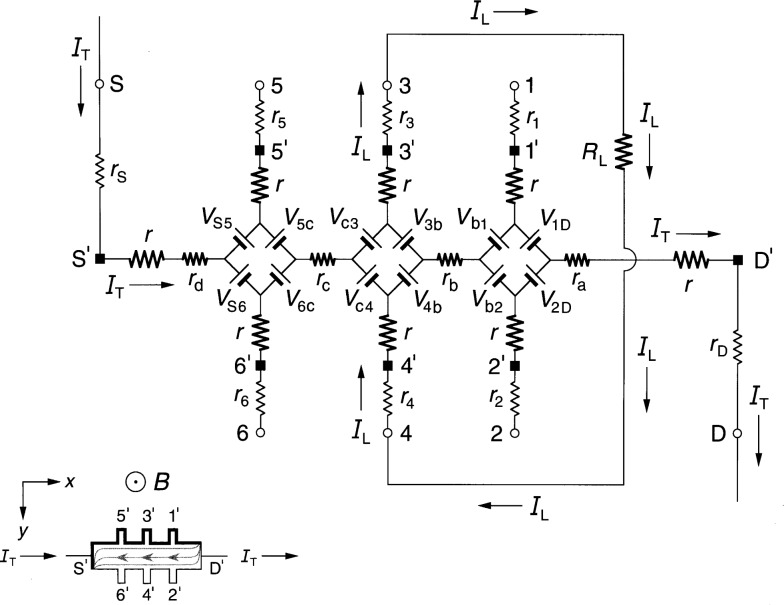
Equivalent circuit using diamond-array voltage generators when a load resistance *R*_L_ is placed across the Hall potential contacts 3 and 4. The results are presented in Sec. 4.1.

Figure 5 shows the equivalent circuit when an external load resistance *R*_L_ is placed across the potential contacts 3 and 4. The directions of the applied current *I*_T_ and the magnetic flux density *B* are reversed from those in Fig. 1 in order to easily compare the results with Ref. [14]. Thus the source contact pad S*′* and the potential contact pads 1*′*, 3*′*, and 5*′* are at a higher potential at this instant in time than the drain contact pad D*′* and the potential contact pads 2*′*, 4*′*, and 6*′* when there is no external load resistor. We have separated the lead resistances *r*_3_ and *r*_4_ from the load resistance *R*_L_. The solutions for the current through the load resistance, the quantum Hall voltages, and the longitudinal voltages are

$$I_{L}=\frac{R_{H}}{\left(R_{H}+R_{L}+r_{3}+r_{4}\right)}\;I_{T}$$ (15)

$$V_{H}(1,2)=V_{H}(5,6)=R_{H}I_{T}$$ (16a)

$$V_{H}(3,4)=R_{H}\left[1-\frac{\left(R_{H}+r_{3}+r_{4}\right)}{\left(R_{H}+R_{L}+r_{3}+r_{4}\right)}\right]\;I_{T}$$ (16b)

and

$$V_{x}(4,2)=\left[r_{b}+\frac{R_{H}(R_{H}+r_{4})}{\left(R_{H}+R_{L}+r_{3}+r_{4}\right)}\right]\;I_{T}$$ (17a)

$$V_{x}(3,1)=\left[r_{b}-\frac{r_{3}R_{H}}{\left(R_{H}+R_{L}+r_{3}+r_{4}\right)}\right]\;I_{T}$$ (17b)

$$V_{x}(6,4)=\left[r_{c}-\frac{r_{4}R_{H}}{\left(R_{H}+R_{L}+r_{3}+r_{4}\right)}\right]\;I_{T}$$ (17c)

$$V_{x}(5,3)=\left[r_{c}+\frac{R_{H}(R_{H}+r_{3})}{\left(R_{H}+R_{L}+r_{3}+r_{4}\right)}\right]\;I_{T}.$$ (17d)

Equation (17b) and Eq. (17c) may explain why some of the *V_x_* measurements of Ricketts and Kemeny [14] were negative when *B* was within a quantized Hall resistance plateau. If this equivalent circuit is a correct representation of quantum Hall devices shunted by load resistors, then negative voltages should be experimentally observable when the second terms within the brackets in these two equations are larger than the longitudinal resistances *r*_b_ and *r*_c_. Even though some measured voltages become negative when an external load resistor is placed across a quantum Hall potential probe set, all resistances in the equivalent circuit remain positive, as they must.

Equations (15) through (17) were derived for an *external* load resistance. If there was *internal* leakage resistance within the device, *R*_L_ would be placed across the contact pads 3*′* and 4*′* in that case, rather than across connection points 3 and 4. The currents then bypass resistances *r*_3_ and *r*_4_, so they would be removed from Eqs. (15) through (17), and there would be no negative longitudinal voltages.

### 4.2 Ring-Array Circuit Results

**Fig. 6:**
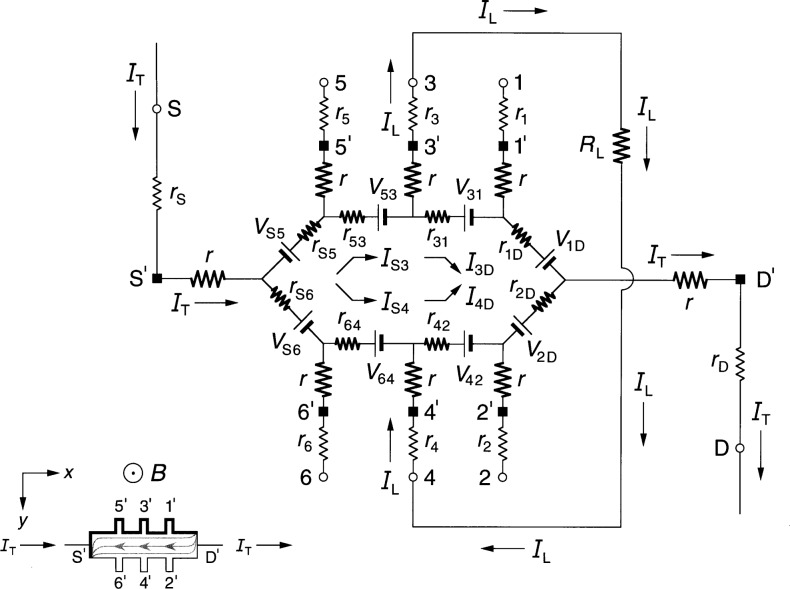
Equivalent circuit using ring-array voltage generators when a load resistance *R*_L_ is placed across the Hall potential contacts 3 and 4. The results are presented in Sec. 4.2.

Figure 6 shows the equivalent circuit with ring-arrays of voltage generators when there is an external load resistance *R*_L_ across the device. We found in Sec. 2.2 that in order to be compatible with the diamond-array results,

$$r_{1D}=r_{2D}=2r_{a}$$ (18a)

$$r_{31}=r_{42}=2r_{b}$$ (18b)

$$r_{53}=r_{64}=2r_{c}$$ (18c)

$$r_{S5}=r_{S6}=2r_{d}.$$ (18d)

It then follows that

$$\begin{array}{l} I_{L}=\frac{R_{H}}{\left(R_{H}+R_{L}+r_{3}+r_{4}\right)}\\ \times \frac{1}{\left[1+\frac{4(r_{a}+r_{b})(r_{c}+r_{d})}{\left(R_{H}+R_{L}+r_{3}+r_{4})(r_{a}+r_{b}+r_{c}+r_{d}\right)}\right]}\;I_{T} \end{array}$$ (19a)

$$\begin{array}{l} I_{\text{S}3}=I_{T}-I_{\text{S}4}=I_{L}+I_{3\text{D}}\\ =\frac{1}{2}\left[1+\frac{2\left(r_{a}+r_{b}\right)}{\left(r_{a}+r_{b}+r_{c}+r_{d}\right)}\right]\;I_{L} \end{array}$$ (19b)

$$I_{\text{S}4}=I_{T}-I_{S3}=I_{4D}-I_{L}=\frac{1}{2}\left[1-\frac{2(r_{a}+r_{b})}{\left(r_{a}+r_{b}+r_{c}+r_{d}\right)}\right]\;I_{L}$$ (19c)

$$V_{H}(1,2)=R_{H}I_{T}-\frac{4r_{a}(r_{c}+r_{d})}{\left(r_{a}+r_{b}+r_{c}+r_{d}\right)}\;I_{L}$$ (20a)

$$V_{H}(5,6)=R_{H}I_{T}+\frac{4r_{d}(r_{a}+r_{b})}{\left(r_{a}+r_{b}+r_{c}+r_{d}\right)}I_{L}.$$ (20b)

Note that, unlike the results derived in Sec. 4.1 using the diamond-array circuit, there are corrections of order 2*r*_a_*I*_L_ to *V*_H_(1,2) and *V*_H_(5,6) in the presence of longitudinal resistance and a load resistor. The exact solution for *V*_H_(3,4) is much more complex than the ring-array Eq. (16b), and we do not give it here; but it can be greatly simplified by making the assumption that the device is homogeneous, so that *r*_a_ + *r*_b_ ≈ *r*_c_ + *r*_d_. Then

$$V_{H}(3,4)\approx R_{H}\left\{1-\frac{\left[R_{H}+r_{3}+r_{4}-2(r_{a}+r_{b})\right]}{\left(R_{H}+R_{L}+r_{3}+r_{4}\right)}\left[1-\frac{2(r_{a}+r_{b})}{\left(R_{H}+R_{L}+r_{3}+r_{4}\right)}\right]\right\}\;I_{T}$$ (21)

and

$$V_{x}(4,2)\approx \left[r_{b}+\frac{R_{H}(R_{H}+r_{4}+2r_{b})}{\left(R_{H}+R_{L}+r_{3}+r_{4}\right)}\right]\;I_{T}$$ (22a)

$$V_{x}(3,1)\approx \left[r_{b}-\frac{(r_{3}+2r_{b})R_{H}}{\left(\;R_{H}+R_{L}+r_{3}+r_{4}\right)}\right]\;I_{T}$$ (22b)

$$V_{x}(6,4)\approx \left[r_{c}-\frac{(r_{4}+2r_{c})\;R_{H}}{\left(R_{H}+R_{L}+r_{3}+r_{4}\right)}\right]I_{T}$$ (22c)

$$V_{x}(5,3)\approx \left[r_{c}+\frac{R_{H}(R_{H}+r_{3}+2r_{c})}{\left(R_{H}+R_{L}+r_{3}+r_{4}\right)}\right]\;I_{T}.$$ (22d)

As with the quantized Hall voltages, the equations for the longitudinal voltages differ somewhat from those of the diamond-array circuit. The numerical values of these expressions are nearly identical for both circuits, however, when typical experimental values are used for the circuit elements.

We have seen in Secs. 2 and 3, and in this section, that in the absence of significant longitudinal resistance, the diamond-array and ring-array circuit results reduce to identical forms. The validity of ring-array circuits with negligible longitudinal resistances present have been verified in the precision experiments of Jeffery, Elmquist, and Cage [16]. We have chosen to use the diamond-shaped voltage generator arrays of Ricketts and Kemeny [14] in the remainder of the calculations, rather than the ring-shaped arrays of Delahaye [13], because the calculations are much easier in the diamond-array circuits when longitudinal resistances are present. Appendix A will, however, give the simplest example of a ring-array multi-series circuit having longitudinal resistance.

## 5. Double-Series Connections

Accurate ac quantized Hall resistance experiments use four-terminal-pair measurement techniques [17,18]. The large coaxial lead resistances and capacitances in the ac sample probes necessitate the use of Delahaye’s [13] multiple-series connections to the device. We use Kirchoff’s rules to sum the currents at branch points and the voltages around loops to obtain exact algebraic solutions for currents and voltages of the equivalent circuits when using multi-series connections to the quantum Hall device. All multi-series connections either used or proposed in the literature are considered.

### 5.1 Hall Voltage Configuration

**Fig. 7:**
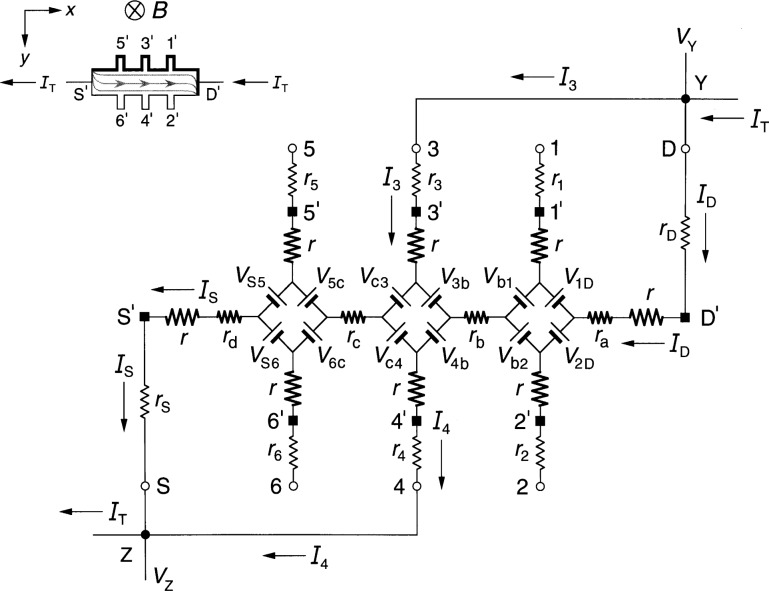
Equivalent circuit for two double-series connections to a quantum Hall effect device with diamond-array voltage generators. The quantized Hall voltage *V*_H_(Y, Z) is measured between points Y and Z. See Sec. 5.1 for the algebraic solutions.

Figure 7 shows two double-series connections to the device. One double-series connection emanates from point Y to points D and 3. The other double-series connection proceeds from point Z to S and 4. Points Y and Z are where the four-terminal-pair definitions [17, 18] of the resistance standard are achieved. *V*_Y_ and *V*_Z_ are voltages at points Y and Z, respectively. The magnetic flux density *B* is directed into the figure, and the total positive current *I*_T_ again enters the device from the right to the left. Therefore the electron flow pattern within the device is the same as that in Fig. 1. The drain contact pad D*′* and potential probe contact pads 1*′*, 3*′*, and 5*′* are all near one potential, while the source contact pad S′ and potential pads 2*′*, 4*′*, and 6*′* are all near another potential.

Inner conductor resistances in the coaxial cables making up the double-series connections are again included in the lead resistances *r*_S_, *r*_1_ through *r*_6_, and *r*_D_ along with the contact resistances to the two-dimensional electron gas and any wire-bonding resistances. The lead resistance of each arm *r*_S_ through *r*_D_ of the equivalent circuit can be determined from pair-wise, two-terminal resistance measurements described in Sec. 2.1. Lead resistances are each typically 1 Ω for ac quantized Hall resistance experiments.

It is important to note that the values for the longitudinal resistances *r*_a_, *r*_b_, *r*_c_, and *r*_d_ shown in Fig. 7 and in all subsequent figures can be obtained from potential difference measurements around the periphery of the device for the *regular* (single-series) connections of Fig. 1. For example, we have *r*_b_ = *R_x_*(2,4) ≡ *V_x_*(2,4)/*I*_T_. The ac longitudinal resistances are of order 1 mΩ at 1592 Hz. We use the regular (single-series) connections to assign values to *r*_a_, *r*_b_, *r*_c_, and *r*_d_ in order to see what errors occur in measurements of multi-series circuits.

The double-series diamond-array circuit current solutions are

$$I_{S}=\frac{\left(R_{H}+r_{4}\right)}{\left(R_{H}+r_{S}+r_{4}+r_{c}+r_{d}\right)}\;I_{T}$$ (23a)

$$I_{D}=\frac{\left(R_{H}+r_{3}\right)}{\left(R_{H}+r_{D}+r_{3}+r_{a}+r_{b}\right)}\;I_{T}$$ (23b)

$$I_{3}=\frac{\left(r_{D}+r_{a}+r_{b}\right)}{\left(R_{H}+r_{D}+r_{3}+r_{a}+r_{b}\right)}\;I_{T}\approx \frac{r_{D}}{R_{H}}\;I_{T}$$ (23c)

$$I_{4}=\frac{\left(r_{S}+r_{c}+r_{d}\right)}{\left(R_{H}+r_{S}+r_{4}+r_{c}+r_{d}\right)}\;I_{T}\approx \frac{r_{S}}{R_{H}}\;I_{T},$$ (23d)

where the values of the quantized Hall resistance *R*_H_ ≡ *V*_H_/*I*_T_ are obtained from single-series quantum Hall voltage measurements. Once again, the values of *R*_H_ should be the same along the device before making double-series connections, but the values are not necessarily equal to the von Klitzing constant *R*_K_.

The quantized Hall voltage measured between points Y and Z is by definition

$$V_{H}(Y,Z)=[V_{Y}-V_{Z}]\equiv R_{H}(Y,Z)\;I_{T}.$$ (24)

Taking the path along potential probes 4 and 3, the quantized Hall voltage is

$$V_{H}(Y,Z)=R_{H}I_{T}+r_{3}I_{3}+r_{4}I_{4}$$ (25a)

$$V_{H}(Y,Z)=R_{H}\left[1+\frac{r_{3}(r_{D}+r_{a}+r_{b})}{R_{H}(R_{H}+r_{D}+r_{3}+r_{a}+r_{b})}+\frac{r_{4}(r_{S}+r_{c}+r_{d})}{R_{H}(R_{H}+r_{S}+r_{4}+r_{c}+r_{d})}\right]\;I_{T},$$ (25b)

or approximately

$$V_{H}(Y,Z)\approx R_{H}\left[1+\frac{r_{3}\;r_{D}}{R_{H}R_{H}}+\frac{r_{4}r_{S}}{R_{H}R_{H}}\right]\;I_{T}.$$ (25c)

The sum of the last two terms within the brackets of Eq. (25b) is the error in measuring *R*_H_(Y,Z) in a double-series circuit relative to the correct single-series circuit value *R*_H_.

Table 1 lists the current ratios and the relative errors in *R*_H_(Y,Z) for four representative cases with double-series connections. The lead resistances in these four cases are all either 1 Ω or 10 Ω, and the longitudinal resistances are all between 0 Ω and 1 mΩ. (We chose 10 Ω because the lead resistances can approach that value in sample probe leads of helium-3 refrigerators.)

There is a fractional correction 1.2 × 10^−8^ to the exact calculation of *R*_H_(Y,Z) when typical 1 Ω ac lead resistances are used in Table 1. This is a *large error* compared with the 2.4 ×10^−8^ relative combined standard uncertainty of the complete measurement chain at NIST [19, 20], i.e., from the quantized Hall resistance to the calculable capacitor. The fractional correction to *R*_H_(Y,Z) should preferably be no larger than 1 ×10^−9^. That is why triple-series or quadruple-series connections are required in accurate experiments.

The approximate solutions to the currents *I*_3_ and *I*_4_, and the quantized Hall resistance *R*_H_(Y,Z) [not shown in the table but given by Eqs. (23c), (23d), and (25c)] are satisfactory. The worst case is for 10 Ω lead resistances and 0.1 mΩ longitudinal resistances where the approximate values of *I*_3_ and *I*_4_ are fractionally 1.2 ×10^−6^ larger than the exact calculation. The approximate value of *R*_H_(Y,Z) is fractionally 1.8 ×10^−9^ larger than the exact calculation for that case.

### 5.2 Longitudinal Voltage Configuration

The most direct method of measuring ac longitudinal voltages is to use the regular single-series connections to the device shown in Fig. 1, where *V_x_*(2,4) = *V_x_*(1,3) ≡ *r*_b_*I*_T_ and *V_x_*(4,6) = *V_x_*(3,5) ≡ *r*_c_*I*_T_. Some experiments, however, report large antenna noise generated on sample probe leads that remain unconnected at potential points 1 through 6. It is reportedly best in that situation to either remove unused potential probe leads at the device potential contact pads 1 ′ through 6 ′, or to connect as many existing sample probe leads as possible to the device.

**Fig. 8:**
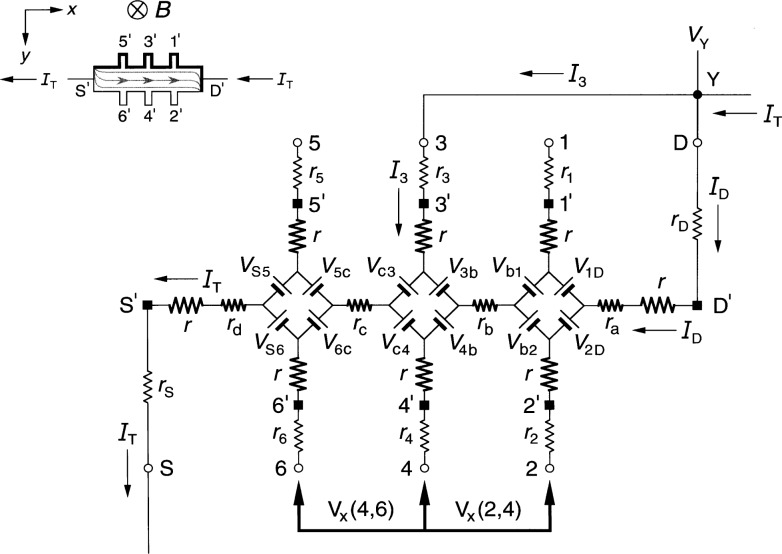
Equivalent circuit for one double-series connection to a quantum Hall effect device with diamond-array voltage generators. The longitudinal Hall voltages *V_x_*(2, 4) and *V_x_*(4, 6) are measured between points 2 and 4, and between points 4 and 6. See Sec. 5.2 for the algebraic solutions.

Removing leads at the contact pads would not be desirable for a resistance standard because the device must be warmed to room temperature between the quantized Hall resistance measurements and the longitudinal resistance measurements; that violates the recommended Consultative Committee on Electricity guide lines [21] for dc quantum Hall effect measurements. Instead, a double-series equivalent circuit shown in Fig. 8 might be used for longitudinal voltage measurements if significant antenna noise is present in the sample probe leads. We assume that potential points 2, 4, and 6 are near virtual ground in the balanced ac bridge, so only open leads 1 and 5 act as antennas. What is the effect on the *V_x_* measurements with this circuit?

$$I_{D}=\frac{\left(R_{H}+r_{3}\right)}{\left(R_{H}+r_{D}+r_{3}+r_{a}+r_{b}\right)}\;I_{T}$$ (26a)

$$I_{3}=\frac{\left(r_{D}+r_{a}+r_{b}\right)}{\left(R_{H}+r_{D}+r_{3}+r_{a}+r_{b}\right)}I_{T}\approx \frac{r_{D}}{R_{H}}I_{T},$$ (26b)

which are the same as in Sec. 5.1, and

$$V_{x}(2,4)=r_{b}\left[1-\frac{\left(r_{D}+r_{a}+r_{b}\right)}{\left(R_{H}+r_{D}+r_{3}+r_{a}+r_{b}\right)}\right]\;I_{T}\approx r_{b}\left[1-\frac{r_{D}}{R_{H}}\right]\;I_{T}$$ (27a)

$$V_{x}(4,6)=r_{c}\;I_{T}.$$ (27b)

There is no correction to *V_x_*(4,6). Table 2 displays the current ratios and the relative errors in *R_x_* (2,4) arising from the three combinations of lead resistances and nonzero longitudinal resistances used in Table 1 for *R*_H_(Y, Z) measurements. The relative errors in *R_x_* (2,4) are acceptably small because *r*_b_ is small; the same argument holds for the approximation to *r*_b_ in Eq. (27a).

## 6. “Normal” Triple-Series Connections

### 6.1 Hall Voltage Configuration

**Fig. 9:**
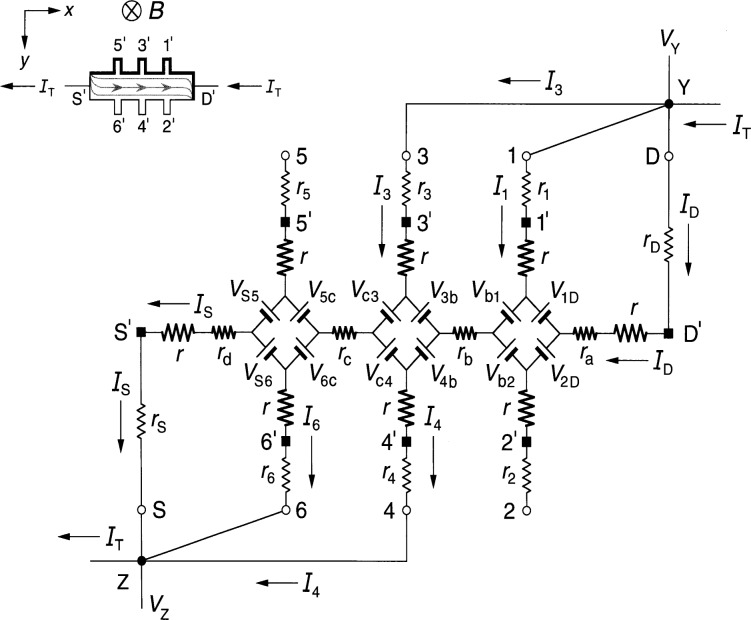
Equivalent circuit for two “normal” triple-series connections to a quantum Hall effect device. The quantized Hall voltage *V*_H_(Y,Z) is measured between points Y and Z. See Sec. 6.1 for the algebraic solutions.

Figure 9 shows two triple-series combinations to the quantum Hall effect device connected in the usual manner. The “normal” triple-series current solutions are

$$I_{3}=\frac{\left[(r_{D}+r_{a})(r_{1}+r_{b})+r_{b}(R_{H}+r_{1})\right]}{\left[(R_{H}+r_{D}+r_{1}+r_{a})(R_{H}+r_{3})+(r_{D}+r_{a})(r_{1}+r_{b})+r_{b}(R_{H}+r_{1})\right]}\;I_{T}$$ (28a)

$$I_{3}\approx \left[\frac{r_{b}}{R_{H}}+\frac{r_{D}r_{1}}{R_{H}R_{H}}\right]\;I_{T}$$ (28b)

$$I_{4}=\frac{\left[(r_{S}+r_{d})(r_{6}+r_{c})+r_{c}(R_{H}+r_{6})\right]}{\left[(R_{H}+r_{S}+r_{6}+r_{d})(R_{H}+r_{4})+(r_{S}+r_{d})(r_{6}+r_{c})+r_{c}(R_{H}+r_{6})\right]}I_{T}$$ (28c)

$$I_{4}\approx \left[\frac{r_{c}}{R_{H}}+\frac{r_{S}r_{6}}{R_{H}R_{H}}\right]\;I_{T}$$ (28d)

$$I_{1}=\frac{\left(r_{D}+r_{a}\right)}{\left(R_{H}+r_{D}+r_{1}+r_{a}\right)}(I_{T}-I_{3})\approx \frac{r_{D}}{R_{H}}I_{T}$$ (28e)

$$I_{6}=\frac{\left(r_{S}+r_{d}\right)}{\left(R_{H}+r_{S}+r_{6}+r_{d}\right)}(I_{T}-I_{4})\approx \frac{r_{S}}{R_{H}}I_{T}$$ (28f)

$$I_{D}=\frac{\left(R_{H}+r_{1}\right)}{\left(r_{D}+r_{a}\right)}I_{1}$$ (28g)

$$I_{S}=\frac{\left(R_{H}+r_{6}\right)}{\left(r_{S}+r_{d}\right)}I_{6}.$$ (28h)

Taking the path along potential probes 4 and 3, the quantized Hall voltage is

$$V_{H}(Y,Z)=R_{H}I_{T}+r_{3}I_{3}+r_{4}I_{4}$$ (29a)

$$\begin{array}{l} V_{H}(Y,Z)=R_{H}\{1+\frac{r_{3}[(r_{D}+r_{a})(r_{1}+r_{b})+r_{b}(R_{H}+r_{1})]}{R_{H}[(R_{H}+r_{D}+r_{1}+r_{a})(R_{H}+r_{3})+(r_{D}+r_{a})(r_{1}+r_{b})+r_{b}(R_{H}+r_{1})]}\\ \;+\frac{r_{4}[(r_{S}+r_{d})(r_{6}+r_{c})+r_{c}(R_{H}+r_{6})]}{R_{H}[(R_{H}+r_{S}+r_{6}+r_{d})(R_{H}+r_{4})+(r_{S}+r_{d})(r_{6}+r_{c})+r_{c}(R_{H}+r_{6})]}\}I_{T}, \end{array}$$ (29b)

or approximately

$$V_{H}(Y,Z)\approx R_{H}\left\{1+\frac{r_{3}[r_{D}r_{1}+r_{b}R_{H}]}{R_{H}R_{H}R_{H}}+\frac{r_{4}[r_{S}r_{6}+r_{c}R_{H}]}{R_{H}R_{H}R_{H}}\right\}I_{T}.$$ (29c)

Table 1 lists the current ratios and the relative errors in *R*_H_(Y,Z) for the four representative cases with “normal” triple-series connections. There is an acceptable fractional 1 × 10^−9^ correction to the exact calculation of *R*_H_(Y,Z) for untypically large 10 Ω ac lead resistances; typical 1 Ω ac lead resistances present no problem at all.

The approximate solutions to the currents [not shown in Table 1 but given by Eqs. (28b), (28d), (28e), and (28f)] are satisfactory. The worst case is for 10 Ω lead resistances and 0.1 mΩ longitudinal resistances, where the approximate values of *I*_1_ and *I*_6_ are fractionally larger by 1.2 × 10^−6^ than the exact calculations. The worst approximation to the value of *R*_H_(Y,Z) [not shown in the table but given by Eq. (29c)] is only fractionally larger by 1.8 × 10^−12^ than the exact calculation for the case of 10 Ω lead resistances and 1 mΩ longitudinal resistances.

### 6.2 Longitudinal Voltage Configuration

**Fig. 10:**
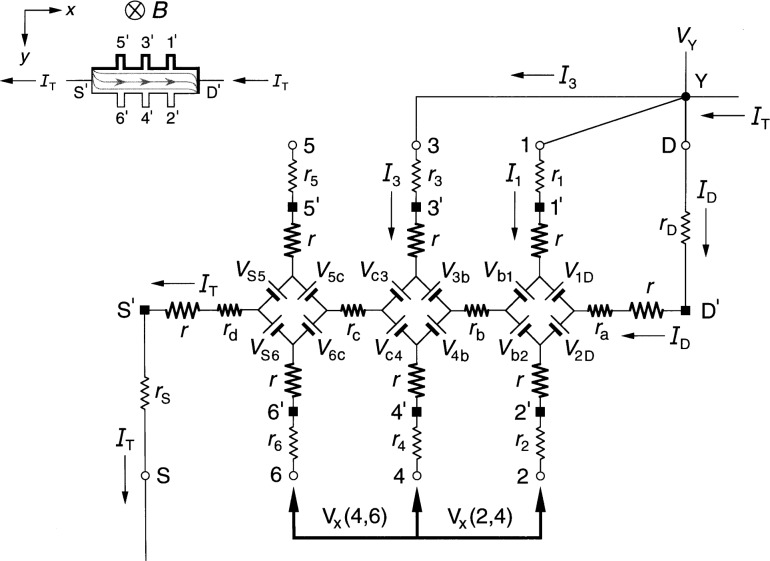
Equivalent circuit for one “normal” triple-series connection to a quantum Hall effect device. The longitudinal Hall voltages *V_x_*(2,4) and *V_x_*(4,6) are measured between points 2, 4 and 4, 6. See Sec. 6.2 for the algebraic solutions.

Figure 10 shows a “normal” triple-series equivalent circuit that can be used for longitudinal voltage measurements if significant sample probe lead antenna noise is present. The solutions for currents *I*_1_, *I*_3_, and *I*_D_ are the same as those listed in Eqs. (28a), (28b), (28e), and (28g). The longitudinal voltages are

$$V_{x}(2,4)=r_{b}I_{T}-r_{b}I_{3}$$ (30a)

$$V_{x}(2,4)=r_{b}\left\{1-\frac{\left[(r_{D}+r_{a})(r_{1}+r_{b})+r_{b}(R_{H}+r_{1})\right]}{\left[(R_{H}+r_{D}+r_{1}+r_{a})(R_{H}+r_{3})+(r_{D}+r_{a})(r_{1}+r_{b})+r_{b}(R_{H}+r_{1})\right]}\right\}I_{T},$$ (30b)

or approximately

$$V_{x}(2,4)\approx r_{b}\left\{1-\left[\frac{r_{b}}{R_{H}}+\frac{r_{D}r_{1}}{R_{H}R_{H}}\right]\right\}I_{T}$$ (30c)

$$V_{x}(4,6)=r_{c}I_{T}.$$ (30d)

There is no correction to *V_x_*(4,6). Table 2 displays the current ratios and the relative errors in *R_x_*(2,4) arising from the three combinations of lead resistances and nonzero longitudinal resistances used in Table 1 for *R*_H_(Y,Z) measurements. The error in *R_x_*(2,4) relative to the correct value *r*_b_, and the approximate value of *R_x_*(2,4) given by Eq. (30c) are acceptably small because *r*_b_ is small.

## 7. “Symmetric” Triple-Series Connections

### 7.1 Hall Voltage Configuration

**Fig. 11:**
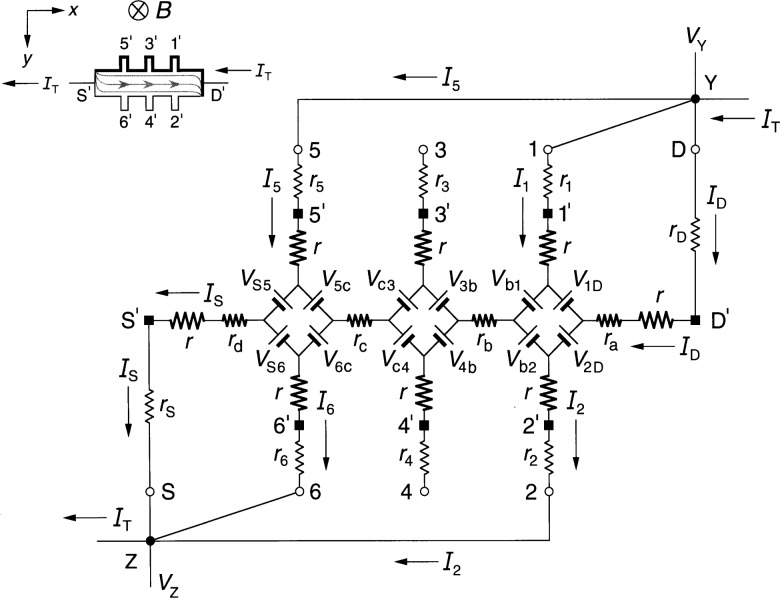
Equivalent circuit for two “symmetric” triple-series connections to a quantum Hall effect device. The quantized Hall voltage *V*_H_(Y,Z) is measured between points Y and Z. See Sec. 7.1 for the algebraic solutions.

Figure 11 shows two triple-series combinations to the quantum Hall effect device connected in a symmetrical manner. The solutions are more complicated, so we define some intermediate substitutions to simplify the final algebraic expressions. Let

$$\hat{a}=[(r_{b}+r_{c})(R_{H}+r_{S}+r_{6}+r_{d})+r_{6}(r_{S}+r_{d})]$$ (31a)

$$\hat{b}=[(r_{b}+r_{c})(R_{H}+r_{S}+r_{6}+r_{d})]$$ (31b)

$$\hat{c}=[(R_{H}+r_{2}+r_{b}+r_{c})(R_{H}+r_{S}+r_{6}+r_{d})+r_{6}(r_{S}+r_{d})]$$ (31c)

$$\hat{d}=[(r_{b}+r_{c})(R_{H}+r_{d}+r_{1}+r_{a})+r_{1}(r_{D}+r_{a})]$$ (31d)

$$\hat{e}=[(r_{b}+r_{c})(R_{H}+r_{D}+r_{1}+r_{a})]$$ (31e)

$$\hat{f}=[(R_{H}+r_{5}+r_{b}+r_{c})(R_{H}+r_{D}+r_{1}+r_{a})+r_{1}(r_{D}+r_{a})].$$ (31f)

The “symmetric” triple-series current solutions are then

$$I_{2}=\left[\frac{(\hat{a}\hat{f}-\hat{b}\hat{d}}{\left(\hat{c}\hat{f}-\hat{b}\hat{e}\right)}\right]I_{T}$$ (32a)

$$I_{2}\approx \left[\frac{\left(r_{b}+r_{c}\right)}{R_{H}}+\frac{r_{S}r_{6}}{R_{H}R_{H}}\right]I_{T}$$ (32b)

$$I_{5}=\left[\frac{\left(\hat{c}\hat{d}-\hat{a}\hat{e}\right)}{\left(\hat{c}\hat{f}-\hat{b}\hat{e}\right)}\right]I_{T}$$ (32c)

$$I_{5}\approx \left[\frac{\left(r_{b}+r_{c}\right)}{R_{H}}+\frac{r_{D}r_{1}}{R_{H}R_{H}}\right]I_{T}$$ (32d)

$$I_{1}=\frac{\left(r_{D}+r_{a}\right)}{\left(R_{H}+r_{D}+r_{1}+r_{a}\right)}(I_{T}-I_{5})\approx \frac{r_{D}}{R_{H}}I_{T}$$ (32e)

$$I_{6}=\frac{\left(r_{S}+r_{d}\right)}{\left(R_{H}+r_{S}+r_{6}+r_{d}\right)}(I_{T}-I_{2})\approx \frac{r_{S}}{R_{H}}I_{T}$$ (32f)

$$I_{D}=\frac{\left(R_{H}+r_{1}\right)}{\left(r_{D}+r_{a}\right)}I_{1}$$ (32g)

$$I_{S}=\frac{\left(R_{H}+r_{6}\right)}{\left(r_{S}+r_{d}\right)}I_{6}.$$ (32h)

Taking the path along potential probes 6 and 5, the quantized Hall voltage is

$$V_{H}(Y,Z)=R_{H}I_{T}-R_{H}I_{2}+r_{5}I_{5}+r_{6}I_{6}$$ (33a)

$$V_{H}(Y,Z)=R_{H}\left\{1-\frac{\left(\hat{a}\hat{f}-\hat{b}\hat{d}\right)}{\left(\hat{c}\hat{f}-\hat{b}\hat{e}\right)}+\frac{r_{5}}{R_{H}}\frac{\left(\hat{c}\hat{d}-\hat{a}\hat{e}\right)}{\left(\hat{c}\hat{f}-\hat{b}\hat{e}\right)}+\frac{r_{6}}{R_{H}}\frac{\left(r_{S}+r_{d}\right)}{\left(R_{H}+r_{S}+r_{6}+r_{d}\right)}\left[1-\frac{\left(\hat{a}\hat{f}-\hat{b}\hat{d}\right)}{\left(\hat{c}\hat{f}-\hat{b}\hat{e}\right)}\right]\right\}I_{T}$$ (33b)

$$V_{H}(Y,Z)\approx R_{H}\left\{1-\frac{\left(r_{b}+r_{c}\right)}{R_{H}}]\right\}I_{T}.$$ (33c)

Table 1 lists the current ratios and the relative errors in *R*_H_(Y,Z) for the four representative cases with “symmetric” triple-series connections. It at first appears that the measured values of *R*_H_(Y,Z) are too small, with fractional errors that can exceed 1.5 × 10^−7^. However, Eq. (33c) predicts that the voltage *V*_H_(Y,Z) measured between points Y and Z is the correct quantized Hall voltage *V*_H_ across the device *minus* the longitudinal voltage *V_x_*(2,6) along the device between points 2 and 6; i.e., that *R*_H_(Y,Z) ≈[*R*_H_ – *R_x_*(2,6)], where *R_x_*(2,6) = *R_x_*(1,5) = [*r*_b_ + *r*_c_]. This prediction for *R*_H_(Y,Z) is within 1 × 10^−9^ of the quantity *R*_H_ – *R_x_*(2,6) when the lead resistances are 10 Ω and is within 3 × 10^−11^ of the same quantity *R*_H_ – *R_x_*(2,6) when the lead resistances are 1 Ω.

The approximate solutions to the currents [not shown in Table 1 but given by Eqs. (32b), (32d), (32e), and (32f)] are satisfactory. The worst case is for 10 Ω lead resistances and 0.1 mΩ longitudinal resistances, where the approximate values of *I*_1_ and *I*_6_ are each fractionally larger than the exact calculations by 1.2 × 10^−6^.

### 7.2 Longitudinal Voltage Configuration

**Fig. 12:**
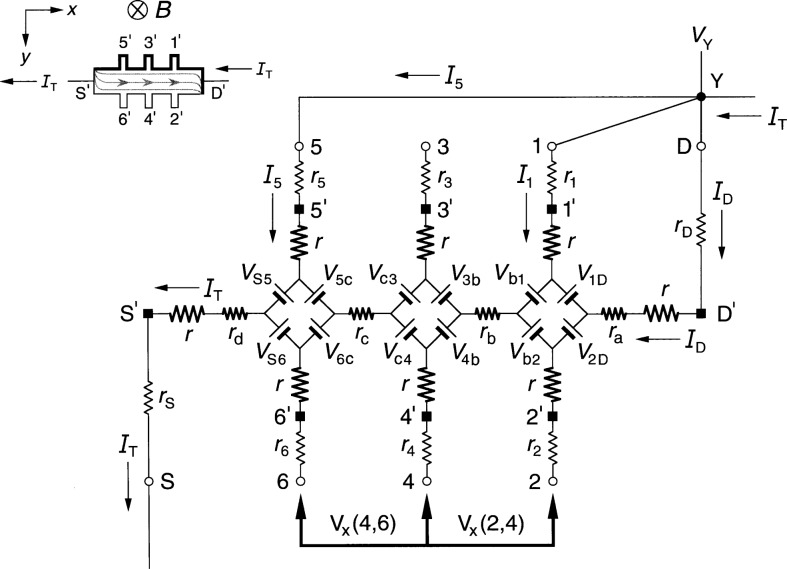
Equivalent circuit for one “symmetric” triple-series connection to a quantum Hall effect device. The longitudinal Hall voltages *V_x_*(2,4) and *V_x_*(4,6) are measured between points 2, 4 and 4, 6. See Sec. 7.2 for the algebraic solutions.

Figure 12 shows a “symmetric” triple-series equivalent circuit that can be used for longitudinal voltage measurements if significant sample probe lead noise is present. The solutions for currents *I*_1_ and *I*_D_ are the same as those listed in Eqs. (32e) and (32g). The solution for *I*_5_ is simpler:

$$I_{5}=\frac{\hat{d}}{\hat{f}}I_{T}=\left\{\frac{\left[(r_{b}+r_{c})(R_{H}+r_{D}+r_{1}+r_{a})+r_{1}(r_{D}+r_{a})\right]}{\left[(R_{H}+r_{5}+r_{b}+r_{c})(R_{H}+r_{D}+r_{1}+r_{a})+r_{1}(r_{D}+r_{a})\right]}\right\}I_{T},$$ (34a)

or approximately

$$I_{5}\approx \left[\frac{\left(r_{b}+r_{c}\right)}{R_{H}}+\frac{r_{D}r_{1}}{R_{H}R_{H}}\right]I_{T}.$$ (34b)

The longitudinal voltages are

$$V_{x}(2,4)=r_{b}I_{T}-r_{b}I_{5}=r_{b}\left[1-\frac{\hat{d}}{\hat{f}}\right]I_{T}$$ (35a)

$$V_{x}(2,4)\approx r_{b}\left\{1-\left[\frac{\left(r_{b}+r_{c}\right)}{R_{H}}+\frac{r_{D}r_{1}}{R_{H}R_{H}}\right]\right\}I_{T}$$ (35b)

$$V_{x}(4,6)=r_{c}I_{T}-r_{c}I_{5}=r_{c}\left[1-\frac{\hat{d}}{\hat{f}}\right]I_{T}$$ (35c)

$$V_{x}(4,6)\approx r_{c}\left\{1-\left[\frac{\left(r_{b}+r_{c}\right)}{R_{H}}+\frac{r_{D}r_{1}}{R_{H}R_{H}}\right]\right\}I_{T}.$$ (35d)

Table 2 displays the current ratios and the relative errors in *R_x_*(2,4) and *R_x_*(4,6) arising from the three combinations of lead resistances and nonzero longitudinal resistances used in Table 1 for *R*_H_(Y,Z) measurements. The current ratio results are identical to those of Sec. 7.1. The errors in *R_x_*(2,4) and *R_x_*(4,6) relative to the correct values *r*_b_ and *r*_c_ are again acceptably small because *r*_b_ and *r*_c_ are small.

## 8. “Offset” Triple-Series Connections

### 8.1 Hall Voltage Configuration

**Fig. 13:**
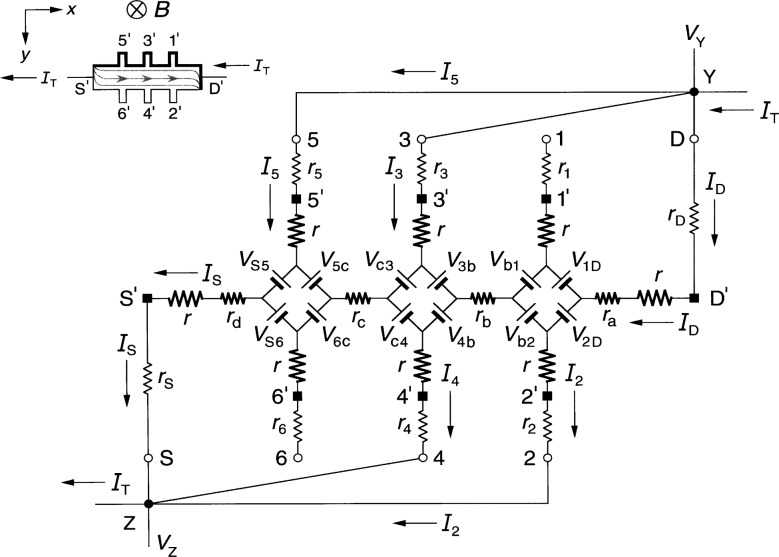
Equivalent circuit for two “offset” triple-series connections to a quantum Hall effect device. The quantized Hall voltage *V*_H_(Y,Z) is measured between points Y and Z. See Sec. 8.1 for the algebraic solutions.

Figure 13 shows two triple-series combinations to the quantum Hall effect device with the connections displaced or “offset” from the symmetric or the normal triple-series configurations. (We consider this case for completeness and because the circuit has been suggested by others.) The solutions are complicated, so once again we define intermediate substitutions to simplify the final algebraic expressions. Let

$$a^{\prime }=\left[r_{b}+\frac{r_{4}(r_{S}+r_{c}+r_{d})}{\left(R_{H}+r_{S}+r_{4}+r_{c}+r_{d}\right)}-\frac{r_{b}(r_{D}+r_{a}+r_{b})}{\left(R_{H}+r_{D}+r_{3}+r_{a}+r_{b}\right)}\right]$$ (36a)

$$b^{\prime }=\left[r_{b}+\frac{r_{4}r_{c}}{\left(R_{H}+r_{S}+r_{4}+r_{c}+r_{d}\right)}-\frac{r_{b}(r_{D}+r_{a}+r_{b})}{\left(R_{H}+r_{D}+r_{3}+r_{a}+r_{b}\right)}\right]$$ (36b)

$$c^{\prime }=\left[(R_{H}+r_{2}+r_{b})+\frac{r_{4}(r_{S}+r_{c}+r_{d})}{\left(R_{H}+r_{S}+r_{4}+r_{c}+r_{d}\right)}-\frac{r_{b}r_{b}}{\left(R_{H}+r_{D}+r_{3}+r_{a}+r_{b}\right)}\right]$$ (36c)

$$d^{\prime }=\left[r_{c}+\frac{r_{3}(r_{D}+r_{a}+r_{b})}{\left(R_{H}+r_{D}+r_{3}+r_{a}+r_{b}\right)}-\frac{r_{c}(r_{S}+r_{c}+r_{d})}{\left(R_{H}+r_{S}+r_{4}+r_{c}+r_{d}\right)}\right]$$ (36d)

$$e^{\prime }=\left[r_{c}+\frac{r_{3}r_{b}}{\left(R_{H}+r_{D}+r_{3}+r_{a}+r_{b}\right)}-\frac{r_{c}(r_{S}+r_{c}+r_{d})}{\left(R_{H}+r_{S}+r_{4}+r_{c}+r_{d}\right)}\right]$$ (36e)

$$f^{\prime }=\left[(R_{H}+r_{5}+r_{c})+\frac{r_{3}(r_{D}+r_{a}+r_{b})}{\left(R_{H}+r_{D}+r_{3}+r_{a}+r_{b}\right)}-\frac{r_{c}r_{c}}{\left(R_{H}+r_{S}+r_{4}+r_{c}+r_{d}\right)}\right].$$ (36f)

The “offset” triple-series current solutions are then

$$I_{2}=\left[\frac{\left(a^{\prime }f^{\prime }-b^{\prime }d^{\prime }\right)}{\left(c^{\prime }f^{\prime }-b^{\prime }e^{\prime }\right)}\right]I_{T}$$ (37a)

$$I_{2}\approx \left[\frac{r_{b}}{R_{H}}+\frac{r_{S}r_{4}}{R_{H}R_{H}}\right]I_{T}$$ (37b)

$$I_{5}=\left[\frac{\left(c^{\prime }d^{\prime }-a^{\prime }e^{\prime }\right)}{\left(c^{\prime }f^{\prime }-b^{\prime }e^{\prime }\right)}\right]I_{T}$$ (37c)

$$I_{5}\approx \left[\frac{r_{c}}{R_{H}}+\frac{r_{D}r_{3}}{R_{H}R_{H}}\right]I_{T}$$ (37d)

$$\begin{array}{l} I_{3}=\frac{\left(r_{D}+r_{a}+r_{b}\right)}{\left(R_{H}+r_{D}+r_{3}+r_{a}+r_{b}\right)}(I_{T}-I_{5})\\ -\frac{r_{b}}{\left(R_{H}+r_{D}+r_{3}+r_{a}+r_{b}\right)}I_{2}\approx \frac{r_{D}}{R_{H}}I_{T} \end{array}$$ (37e)

$$\begin{array}{l} I_{4}=\frac{\left(r_{S}+r_{c}+r_{d}\right)}{\left(R_{H}+r_{S}+r_{4}+r_{c}+r_{d}\right)}(I_{T}-I_{2})\\ -\frac{r_{c}}{\left(R_{H}+r_{S}+r_{4}+r_{c}+r_{d}\right)}I_{5}\approx \frac{r_{S}}{R_{H}}I_{T} \end{array}$$ (37f)

$$I_{D}=\frac{\left(R_{H}+r_{3}\right)}{\left(r_{D}+r_{a}+r_{b}\right)}I_{3}+\frac{r_{b}}{\left(r_{D}+r_{a}+r_{b}\right)}I_{2}$$ (37g)

$$I_{S}=\frac{\left(R_{H}+r_{4}\right)}{\left(r_{S}+r_{c}+r_{d}\right)}I_{4}+\frac{r_{c}}{\left(r_{S}+r_{c}+r_{d}\right)}I_{5}.$$ (37h)

Taking the path along potential probes 4 and 3, we find the quantized Hall voltage is

$$V_{H}(Y,Z)=R_{H}I_{T}-R_{H}(I_{2}+I_{5})+r_{3}\;I_{3}+r_{4}I_{4},$$ (38a)

or approximately

$$V_{H}(Y,Z)\approx R_{H}\left\{1-\left[\frac{\left(r_{b}+r_{c}\right)}{R_{H}}\right]\right\}I_{T}.$$ (38b)

Table 1 lists the current ratios and the relative errors in *R*_H_(Y,Z) for the four representative cases with “offset” triple-series connections. The measured values of *R*_H_(Y,Z) would again be too small, with errors that are identical to the “symmetric” triple-series configuration. However, Eq. (38b) predicts that the voltage *V*_H_(Y,Z) measured between points Y and Z is once again the correct quantized Hall voltage *V*_H_ across the device minus the longitudinal voltage *V_x_*(2,6) along the device between points 2 and 6; i.e., that *R*_H_(Y,Z) ≈ [*R*_H_ – *R_x_*(2,6)], where *R_x_*(2,6) = *R_x_*(1,5) = [*r*_b_ + *r*_c_]. This prediction for *R*_H_(Y,Z) is again within 1 × 10^−9^ of the quantity *R*_H_ – *R_x_*(2,6) when the lead resistances are 10 Ω, and is within 3 × 10^−11^ of the same quantity when the lead resistances are 1 Ω.

The approximate solutions to the currents [not shown in Table 1 but given by Eqs. (37b), (37d), (37e), and (37f)] are satisfactory. The worst case is for 10 Ω lead resistances and 0.1 mΩ longitudinal resistances, where the approximate values of *I*_3_ and *I*_4_ are fractionally larger than the exact calculations by 1.2 × 10^−6^.

### 8.2 Longitudinal Voltage Configuration

**Fig. 14:**
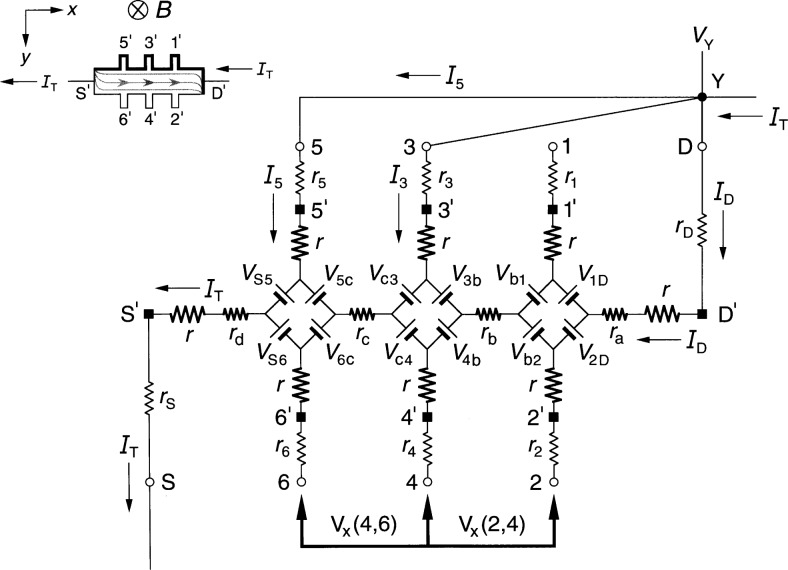
Equivalent circuit for one “offset” triple-series connection to a quantum Hall effect device. The longitudinal Hall voltages *V_x_*(2,4) and *V_x_*(4,6) are measured between points 2, 4 and 4, 6. See Sec. 8.2 for the algebraic solutions.

Figure 14 shows an “offset” triple-series equivalent circuit that can be used for longitudinal voltage measurements if significant sample probe lead noise is present. The solutions for the currents are simpler than in Sec. 8.1:

$$I_{5}=\left\{\frac{\left[r_{c}+\frac{r_{3}(r_{D}+r_{a}+r_{b})}{\left(R_{H}+r_{D}+r_{3}+r_{a}+r_{b}\right)}\right]}{\left[(R_{H}+r_{5}+r_{c})+\frac{r_{3}(r_{D}+r_{a}+r_{b})}{\left(R_{H}+r_{D}+r_{3}+r_{a}+r_{b}\right)}\right]}\right\}I_{T}\approx \frac{d^{\prime }}{f^{\prime }}I_{T}$$ (39a)

$$I_{5}\approx \left[\frac{r_{c}}{R_{H}}+\frac{r_{D}r_{3}}{R_{H}R_{H}}\right]I_{T}$$ (39b)

$$I_{3}=\frac{\left(r_{D}+r_{a}+r_{b}\right)}{\left(R_{H}+r_{D}+r_{3}+r_{a}+r_{b}\right)}(I_{T}-I_{5})\approx \frac{r_{D}}{R_{H}}I_{T}$$ (39c)

$$I_{D}=\frac{\left(R_{H}+r_{3}\right)}{\left(r_{D}+r_{a}+r_{b}\right)}I_{3}.$$ (39d)

The longitudinal voltages are

$$V_{x}(2,4)=r_{b}I_{T}-r_{b}I_{3}-r_{b}I_{5}$$ (40a)

$$V_{x}(2,4)\approx r_{b}\left\{1-\left[\frac{r_{D}}{R_{H}}+\frac{r_{c}}{R_{H}}+\frac{r_{D}r_{3}}{R_{H}R_{H}}\right]\right\}I_{T}$$ (40b)

$$V_{x}(4,6)=r_{c}I_{T}-r_{c}I_{5}$$ (40c)

$$V_{x}(4,6)\approx r_{c}\left\{1-\left[\frac{r_{c}}{R_{H}}+\frac{r_{D}r_{3}}{R_{H}R_{H}}\right]\right\}I_{T}.$$ (40d)

Table 2 displays the current ratios and the relative errors in *R_x_*(2,4) and *R_x_*(4,6) arising from the three combinations of lead resistances and nonzero longitudinal resistances used in Table 1 for *R*_H_(Y,Z) measurements. The current ratio results are nearly identical to those of Sec. 8.1. The errors in *R_x_*(2,4) are larger than *R_x_*(4,6), but both errors are acceptably small because *r*_b_ and *r*_c_ are small.

## 9. Quadruple-Series Connections

### 9.1 Hall Voltage Configuration

**Fig. 15:**
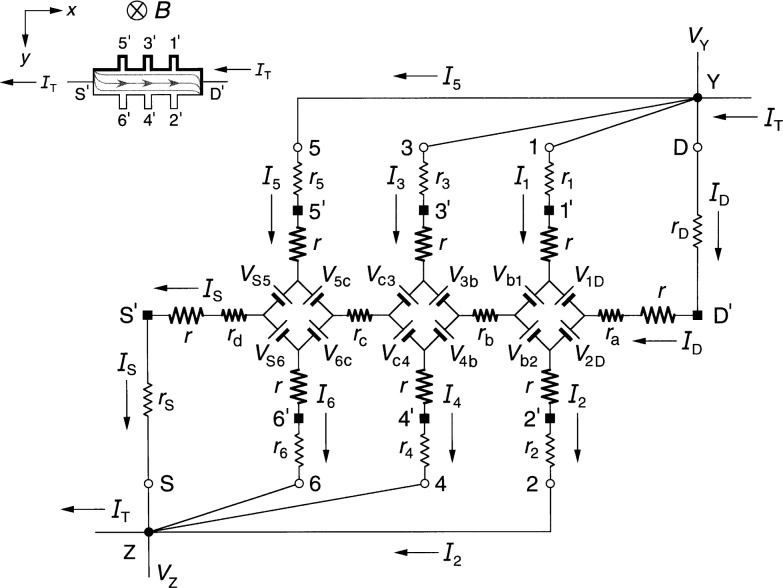
Equivalent circuit for two quadruple-series connections to a quantum Hall effect device. The quantized Hall voltage *V*_H_(Y,Z) is measured between points Y and Z. See Sec. 9.1 for the algebraic solutions.

Figure 15 shows two quadruple-series combinations to the quantum Hall effect device. The solutions are even more complicated, so we define substitutions of substitutions to simplify the final exact algebraic expressions. Let

$$\hat{m}=(R_{H}+r_{2}+r_{b})$$ (41a)

$$\hat{n}=(R_{H}+r_{5}+r_{c})$$ (41b)

$$\hat{o}=[(r_{S}+r_{d})(r_{6}+r_{c})+r_{c}(R_{H}+r_{6})]$$ (41c)

$$\hat{p}=[r_{c}(R_{H}+r_{S}+r_{6}+r_{d})]$$ (41d

$$\hat{q}=[(R_{H}+r_{S}+r_{6}+r_{d})(R_{H}+r_{4})+(r_{S}+r_{d})(r_{6}+r_{c})+r_{c}(R_{H}+r_{6})]$$ (41e)

$$\hat{s}=[(r_{D}+r_{a})(r_{1}+r_{b})+r_{b}(R_{H}+r_{1})]$$ (41f)

$$\hat{t}=[r_{b}(R_{H}+r_{D}+r_{1}+r_{a})]$$ (41g)

$$\hat{u}=[(R_{H}+r_{D}+r_{1}+r_{a})(R_{H}+r_{3})+(r_{D}+r_{a})(r_{1}+r_{b})+r_{b}(R_{H}+r_{1})]$$ (41h)

$$\hat{v}=[r_{b}\hat{q}(\hat{u}-\hat{s})]$$ (41i)

$$\hat{w}=[\hat{q}(\hat{m}\hat{u}-r_{b}\hat{t})]$$ (41j)

$$\hat{y}=[r_{c}\hat{u}(\hat{q}-\hat{o})]$$ (41k)

$$\hat{z}=[\hat{u}(\hat{n}\hat{q}-r_{c}\hat{p})].$$ (41l)

The quadruple-series exact and approximate current solutions are then

$$I_{2}=\left\{\frac{\left[(\hat{v}+r_{4}\hat{o}\hat{u})(\hat{z}+r_{3}\hat{q}s)-(\hat{v}+r_{4}\hat{p}\hat{u})(\hat{y}+r_{3}\hat{q}\hat{s})\right]}{\left[(\hat{w}+r_{4}\hat{o}\hat{u})(\hat{z}+r_{3}\hat{q}\hat{s})-(\hat{v}+r_{4}\hat{p}\hat{u})(\hat{y}+r_{3}\hat{q}\hat{t})\right]}\right\}I_{T}$$ (42a)

$$I_{2}\approx \frac{r_{b}}{R_{H}}I_{T}$$ (42b)

$$I_{5}=\left\{\frac{\left[(\hat{w}+r_{4}\hat{o}\hat{u})(\hat{y}+r_{3}\hat{q}\hat{s})-(\hat{v}+r_{4}\hat{o}\hat{u})(\hat{y}+r_{3}\hat{q}\hat{t})\right]}{\left[(\hat{w}+r_{4}\hat{o}\hat{u})(\hat{z}+r_{3}\hat{q}\hat{s})-(\hat{v}+r_{4}\hat{p}\hat{u})(\hat{y}+r_{3}\hat{q}\hat{t})\right]}\right\}I_{T}$$ (42c)

$$I_{5}\approx \frac{r_{c}}{R_{H}}I_{T}$$ (42d)

$$I_{3}=\frac{\hat{s}}{\hat{u}}I_{T}-\frac{\hat{t}}{\hat{u}}I_{2}-\frac{\hat{s}}{\hat{u}}I_{5}$$ (42e)

$$I_{3}\approx \left[\frac{r_{b}}{R_{H}}+\frac{r_{D}r_{1}}{R_{H}R_{H}}\right]I_{T}$$ (42f)

$$I_{4}=\frac{\hat{o}}{\hat{q}}I_{T}-\frac{\hat{o}}{\hat{q}}I_{2}-\frac{\hat{p}}{\hat{q}}I_{5}$$ (42g)

$$I_{4}\approx \left[\frac{r_{c}}{R_{H}}+\frac{r_{S}r_{6}}{R_{H}R_{H}}\right]I_{T}$$ (42h)

$$I_{1}=\frac{\left(r_{D}+r_{a}\right)}{\left(R_{H}+r_{D}+r_{1}+r_{a}\right)}(I_{T}-I_{3}-I_{5})\approx \frac{r_{D}}{R_{H}}I_{T}$$ (42i)

$$I_{6}=\frac{\left(r_{S}+r_{d}\right)}{\left(R_{H}+r_{S}+r_{6}+r_{d}\right)}(I_{T}-I_{2}-I_{4})\approx \frac{r_{S}}{R_{H}}I_{T}$$ (42j)

$$I_{D}=\frac{\left(R_{H}+r_{1}\right)}{\left(r_{D}+r_{a}\right)}I_{1}$$ (42k)

$$I_{S}=\frac{\left(R_{H}+r_{6}\right)}{\left(r_{S}+r_{d}\right)}I_{6}.$$ (42l)

Taking the path along potential probes 4 and 3, the quantized Hall voltage is

$$V_{H}(Y,Z)=R_{H}I_{T}-R_{H}(I_{2}+I_{5})+r_{3}I_{3}+r_{4}I_{4},$$ (43a)

or approximately

$$V_{H}(Y,Z)\approx R_{H}\left\{1-\left[\frac{\left(r_{b}+r_{c}\right)}{R_{H}}\right]\right\}I_{T}.$$ (43b)

Table 1 lists the current ratios and the relative errors in *R*_H_(Y,Z) for the four representative cases with quadruple-series connections. The measured values of *R*_H_(Y,Z) would again be too small, with errors that are nearly identical to the “symmetric” and “offset” triple-series configurations. However, Eq. (43b) once again predicts that the voltage *V*_H_(Y,Z) measured between points Y and Z is the correct quantized Hall voltage *V*_H_ across the device minus the longitudinal voltage *V_x_*(2,6) along the device between points 2 and 6; i.e., that *R*_H_(Y,Z) ≈ [*R*_H_ – *R_x_*(2,6)], where *R_x_*(2,6) = *R_x_*(1,5) = [*r*_b_ + *r*_c_]. This prediction for *R*_H_(Y,Z) is within 1.2 × 10^−10^ of the quantity *R*_H_ – *R_x_*(2,6) when the lead resistances are 10 Ω.

The approximate solutions to the currents [not shown in Table 1 but given by Eqs. (42b), (42d), (42f), (42h), (42i), and (42j)] are satisfactory. The worst case is for 10 Ω lead resistances and 0.1 mΩ longitudinal resistances, where the approximate values of *I*_1_ and *I*_6_ are each fractionally larger than the exact calculations by 1.2 × 10^−6^.

### 9.2 Longitudinal Voltage Configuration

**Fig. 16:**
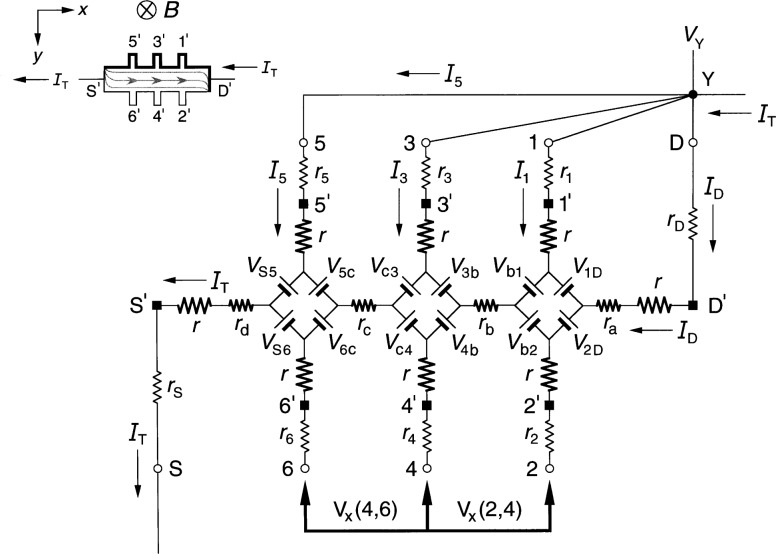
Equivalent circuit for one quadruple-series connection to a quantum Hall effect device. The longitudinal Hall voltages *V_x_*(2,4) and *V_x_*(4,6) are measured between points 2, 4 and 4, 6. See Sec. 9.2 for the algebraic solutions.

Figure 16 shows a quadruple-series equivalent circuit that can be used for longitudinal voltage measurements if significant sample probe lead noise is present. The solutions for the currents are much simpler than in Sec. 9.1:

$$I_{5}=\frac{\left[r_{c}\hat{u}+r_{3}\hat{s}\right]}{\left[\hat{n}\hat{u}+r_{3}\hat{s}\right]}I_{T}$$ (44a)

$$I_{5}\approx \frac{r_{c}}{R_{H}}I_{T}$$ (44b)

$$I_{3}=\frac{\hat{s}}{\hat{u}}(I_{T}-I_{5})$$ (44c)

$$I_{3}\approx \left[\frac{r_{b}}{R_{H}}+\frac{r_{D}r_{1}}{R_{H}R_{H}}\right]I_{T}$$ (44d)

$$I_{1}=\frac{\left(r_{D}+r_{a}\right)}{\left(R_{H}+r_{D}+r_{1}+r_{a}\right)}(I_{T}-I_{3}-I_{5})\approx \frac{r_{D}}{R_{H}}I_{T}$$ (44e)

$$I_{D}=\frac{\left(R_{H}+r_{1}\right)}{\left(r_{D}+r_{a}\right)}I_{1}.$$ (44f)

The longitudinal voltages are

$$V_{x}(2,4)=r_{b}I_{T}-r_{b}I_{3}-r_{b}I_{5}$$ (45a)

$$V_{x}(2,4)=r_{b}\left\{\left[1-\frac{\hat{s}}{\hat{u}}\right]\left[1-\frac{\left(r_{c}\hat{u}+r_{3}\hat{s}\right)}{\left(\hat{n}\hat{u}+r_{3}\hat{s}\right)}\right]\right\}I_{T}$$ (45b)

$$V_{x}(2,4)\approx r_{b}\left\{1-\left[\frac{\left(r_{b}+r_{c}\right)}{R_{H}}+\frac{r_{D}r_{1}}{R_{H}R_{H}}\right]\right\}I_{T}$$ (45c)

$$V_{x}(4,6)=r_{c}I_{T}-r_{c}I_{5}$$ (45d)

$$V_{x}(4,6)=r_{c}\left\{1-\frac{\left(r_{c}\hat{u}+r_{3}\hat{s}\right)}{\left(\hat{n}\hat{u}+r_{3}\hat{s}\right)}\right\}I_{T}$$ (45e)

$$V_{x}(4,6)\approx r_{c}\left[1-\frac{r_{c}}{R_{H}}\right]I_{T}.$$ (45f)

Table 2 displays the current ratios and the relative errors in *R_x_*(2,4) and *R_x_*(4,6) arising from the three combinations of lead resistances and nonzero longitudinal resistances used in Table 1 for *R*_H_(Y,Z) measurements. The current ratio results are identical to those of Sec. 9.1. The errors in *R_x_*(2,4) and *R_x_*(4,6) are very small because *r*_b_ and *r*_c_ are very small.

## 10. Conclusions

We have derived the exact algebraic solutions (and approximate solutions) for a variety of multi-series connections to equivalent electrical circuits of quantum Hall effect devices which have significant longitudinal resistances. All of the circuit element resistances can be determined experimentally from single-series voltage measurements around the periphery of the device. The approximate solutions are adequate for the representative cases we have considered, but it is preferable for the reader to use the exact solutions when applying corrections to their experimental results.

We have found that, in all the circuits considered, the current in each external arm of a circuit is nearly identical for the longitudinal voltage configuration (with one multi-series connection to the device) and for the quantized Hall voltage configuration (with two multi-series connections to the device). Since it is much easier to derive the current equations for a single multi-series connection, it may be safe to use a single multi-series connection configuration when deriving current equations in other circuits not considered here. Although the diamond-shaped voltage generator arrays and the ring-shaped voltage generator arrays both give similar results when including longitudinal resistances, it is much easier to derive the equations using circuits with diamond-shaped voltage generator arrays. Also, the diamond-array solutions are simpler. (Compare the results in Sec. 5 and Appendix A as an example.) Thus, we recommend using diamond-array equivalent circuits.

It is preferable to measure the longitudinal voltages with regular single-series connections to the device (as in Fig. 1). However, if antenna noise generated in the sample probe leads becomes a problem, or if the impedances of the coaxial sample probe leads are too large, then a single quadruple-series connection to the device (as in Fig. 16) is preferable when making ac longitudinal voltage measurements because all the off-ground potential leads are connected in that configuration. A single “normal,” “symmetric,” or “offset” triple-series connection could be used for ac longitudinal voltage measurements if one sample probe potential lead was not connected at device contacts 1′, 3′, or 5′.

Not surprisingly, the largest multi-series errors in *R*_H_(Y,Z) occur for double-series connections. Triple-series or quadruple-series connections should be used for accurate quantized Hall resistance measurements. However, the double-series errors calculated here are still an order of magnitude smaller than the experimentally observed deviations of the ac values of *R*_H_(Y,Z) from the dc values of *R*_H_(Y,Z) when using typical 1 Ω sample probe lead resistances. The multi-series connection errors calculated here are due mainly to the lead resistances [13, 16]. We can see from Table 1 that the multi-series connection errors are insensitive to the longitudinal resistances, except for the triple-series “symmetric,” triple-series “offset,” and the quadruple-series connections which measure the quantity *R*_H_(Y,Z) ≈ [*R*_H_ – *R_x_*(2,6)].

The exact algebraic solutions of the equivalent circuits presented here can be used with confidence to make corrections to dc measurements when using multi-series connections to quantum Hall effect devices. These dc corrections should be used when comparing the dc and ac values of the quantized Hall resistances. However, another purpose of this work is to begin investigating the effect of ac longitudinal resistances on measurements of ac quantized Hall resistances. Finding exact solutions to the complete multi-series ac circuits in the presence of finite ac longitudinal resistances is an extremely difficult problem because the circuits should ultimately include all of the inner conductor-to-shield capacitances, all of the inner conductor-to-inner conductor capacitances, and all of the inductances of the device, the device holder, and the sample probe. As a first step in solving the complete circuit we have ignored these capacitances and inductances, and have considered only the contributions of ac longitudinal resistances on the ac quantized Hall resistance measurements of multiply-connected devices. We find that finite ac longitudinal resistances within the devices do not explain the observed frequency dependences of the ac quantized Hall resistances, i.e., the frequency dependences of the ac quantized Hall resistances are not due to ac longitudinal resistances.

## Figures and Tables

**Table 1:** Current ratios and fractional errors in multi-series measurements of the quantized Hall resistance *R*_H_(Y,Z) when all the lead resistances have representative values *r*_D_ and all the longitudinal resistances have representative values *r*_b_

Configuration	Fig./Sec. numbers	Lead resistance *r*_D_ (Ω)	Longitudinal resistance *r*_b_ (mΩ)	Current ratio *I*_1_/*I*_T_	Current ratio *I*_3_/*I*_T_	Current ratio *I*_5_/*I*_T_	Fractional Hall resistance error [*R*_H_(Y,Z)/*R*_H_] –1
Double-series	7/5.1	10	1.0		7.738 × 10^−4^		1.199 × 10^−6^
		10	0.1		7.736 × 10^−4^		1.199 × 10^−6^
		1	1.0		7.762 × 10^−5^		1.203 × 10^−8^
		1	0		7.747 × 10^−5^		1.200 × 10^−8^
Triple-series “normal”	9/6.1	10	1.0	7.737 × 10^−4^	6.764 × 10^−7^		1.048 × 10^−9^
		10	0.1	7.736 × 10^−4^	6.067 × 10^−7^		9.401 × 10^−10^
		1	1.0	7.755 × 10^−5^	8.348 × 10^−8^		1.294 × 10^−11^
		1	0	7.747 × 10^−5^	6.002× 10^−9^		9.301 × 10^−13^
Triple-series “symmetric”	11/7.1	10	1.0	7.737 × 10^−4^		7.538 × 10^−7^	–1.538 × 10^−7^ a
		10	0.1	7.736 × 10^−4^		6.144 × 10^−7^	–1.454 × 10^−8^ a
		1	1.0	7.755 × 10^−5^		1.610 × 10^−7^	–1.549 × 10^−7^ a
		1	0	7.747 × 10^−5^		6.002 × 10^−9^	9.301 × 10^−13^
Triple-series “offset”	13/8.1	10	1.0		7.738 × 10^−4^	6.764 × 10^−7^	–1.538 × 10^−7^ b
		10	0.1		7.736 × 10^−4^	6.067 × 10^−7^	–1.454 × 10^−8^ b
		1	1.0		7.762 × 10^−5^	8.348 × 10^−8^	–1.549 × 10^−7^ b
		1	0		7.747 × 10^−5^	6.002× 10^−9^	9.301 × 10^−13^
Quad-series	15/9.1	10	1.0	7.737 × 10^−4^	6.764 × 10^−7^	7.794 × 10^−8^	–1.548 × 10^−7^ c
		10	0.1	7.736 × 10^−4^	6.067 × 10^−7^	8.212 × 10^−9^	–1.548 × 10^−8^ c
		1	1.0	7.755 × 10^−5^	8.348 × 10^−8^	7.748 × 10^−8^	–1.550 × 10^−7^ c
		1	0	7.747 × 10^−5^	6.002 × 10^−9^	4.650 × 10^−13^	0

a Eq. (33c) predicts that the measured quantized Hall resistance *R*_H_(Y,Z) is approximately the quantized Hall resistance *R*_H_ *minus* the longitudinal resistance (*r*_b_ + *r*_c_), rather than just *R*_H_.

b Eq. (38b) predicts that the measured quantized Hall resistance *R*_H_(Y,Z) is approximately the quantized Hall resistance *R*_H_ *minus* the longitudinal resistance (*r*_b_ + *r*_c_), rather than just *R*_H_.

c Eq. (43b) predicts that the measured quantized Hall resistance *R*_H_(Y,Z) is approximately the quantized Hall resistance *R*_H_ *minus* the longitudinal resistance (*r*_b_ + *r*_c_), rather than just *R*_H_.

**Table 2:** Current ratios and fractional errors in multi-series measurements of the longitudinal resistances *R_x_*(2,4) and *R_x_*(4,6) when all the lead resistances have representative values *r*_D_ and all the longitudinal resistances have representative values *r*_b_

Configuration	Fig./Sec. numbers	Lead resist. *r*_D_ (Ω)	Long. resist. *r*_b_ (mΩ)	Current ratio *I*_1_/*I*_T_	Current ratio *I*_3_/*I*_T_	Current ratio *I*_5_/*I*_T_	Fractional resistance error *R_x_*(2,4)/*r*_b_–1	Fractional resistance error *R_x_*(4,6)/*r*_c_–1
Double-series	8/5.2	10	1.0		7.738 × 10^−4^		–7.738 × 10^−4^	0
		10	0.1		7.736 × 10^−4^		–7.736 × 10^−4^	0
		1	1.0		7.762 × 10^−5^		–7.762 × 10^−5^	0
Triple-series “normal”	10/6.2	10	1.0	7.737 × 10^−4^	6.764 × 10^−7^		–6.764 × 10^−7^	0
		10	0.1	7.736 × 10^−4^	6.067 × 10^−7^		–6.067 × 10^−7^	0
		1	1.0	7.755 × 10^−5^	8.348 × 10^−8^		–8.348 × 10^−8^	0
Triple-series “symmetric”	12/7.2	10	1.0	7.737 × 10^−4^		7.538 × 10^−7^	–7.538 × 10^−7^	–7.538 × 10^−7^
		10	0.1	7.736 × 10^−4^		6.144 × 10^−7^	–6.144 × 10^−7^	–6.144 × 10^−7^
		1	1.0	7.755 × 10^−5^		1.610 × 10^−7^	–1.610 × 10^−7^	–1.610 × 10^−7^
Triple-series “offset”	14/8.2	10	1.0		7.738 × 10^−4^	6.765 × 10^−7^	–7.744 × 10^−4^	–6.764 ×10^−7^
		10	0.1		7.736 × 10^−4^	6.067 × 10^−7^	–7.742 × 10^−4^	–6.607 × 10^−7^
		1	1.0		7.762 × 10^−5^	8.349 × 10^−8^	–7.771 × 10^−5^	–8.348 × 10^−8^
Quad-series	16/9.2	10	1.0	7.737 × 10^−4^	6.764 × 10^−7^	7.794 × 10^−8^	–7.544 × 10^−7^	–7.794 × 10^−8^
		10	0.1	7.736 × 10^−4^	6.067 × 10^−7^	8.212 × 10^−9^	–6.149 × 10^−7^	–8.212 × 10^−9^
		1	1.0	7.755 × 10^−5^	8.348 × 10^−8^	7.748 × 10^−8^	–1.610 × 10^−7^	–7.748 × 10^−8^

**Fig. A-1:**
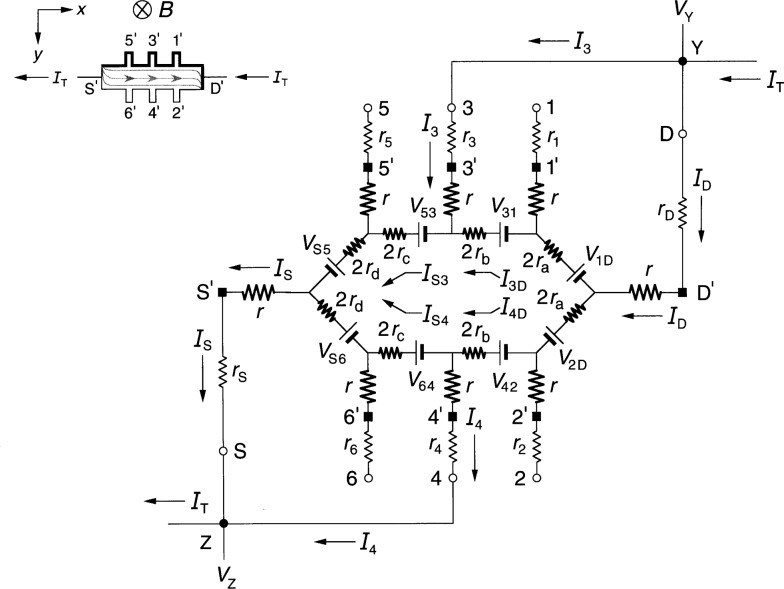
Equivalent circuit for two double-series connections to a quantum Hall effect device with ring-array voltage generators. The quantized Hall voltage *V*_H_(Y,Z) is measured between points Y and Z. See Sec. 11.1 for the algebraic solutions.

**Fig. A-2:**
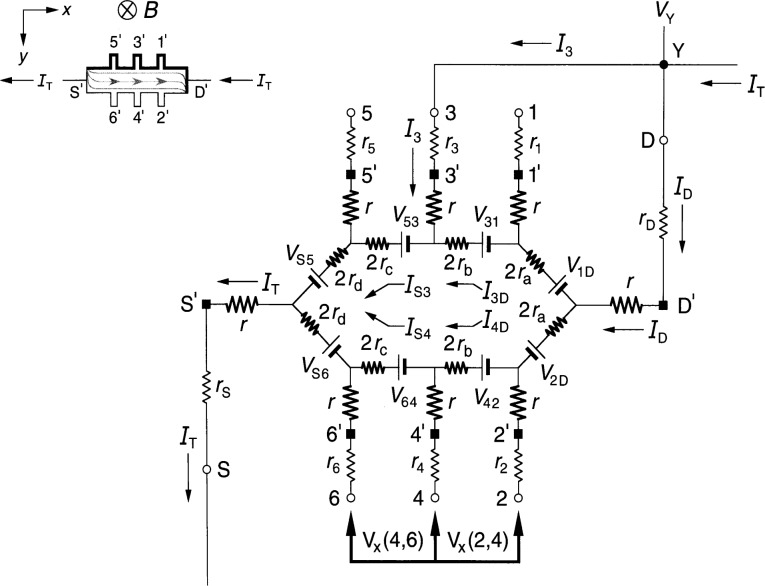
Equivalent circuit for one quadruple-series connection to a quantum Hall effect device. The longitudinal Hall voltages *V_x_*(2,4) and *V_x_*(4,6) are measured between points 2, 4 and 4, 6. See Sec. 11.2 for the algebraic solutions.

## 11. Appendix A. Ring-Array Double-Series Connections

This appendix demonstrates the complexity of exact solutions for ring-array multi-series connections compared with the solutions for diamond-arrays when longitudinal resistances are included in the circuit. We consider only the simplest case (double-series connections), which can be compared with the double-series diamond-array solutions of Sec. 5.

Because the ring-array solutions are so complex, and the numerical results differ insignificantly from the diamond-array circuit results, we use the diamond-array circuits in the main text to find the solutions for the currents, quantized Hall voltages, and longitudinal voltages of triple-series and quadruple-series connections to the device. It is left as an exercise for the reader to find the exact ring-array solutions for triple-series and quadruple-series connections.

### 11.1 Hall Voltage Conriguration

Figure A-1 shows two double-series connections to the device. We define four internal currents *I*_S3_, *I*_S4_, *I*_3D_, and *I*_4D_, and make the following substitutions to simplify the current and voltage equations:

$$\hat{A}=\left\{\frac{r_{D}}{\left[R_{H}+r_{D}+r_{3}+2(r_{a}+r_{b})\right]}\right\}$$ (A-1a)

$$\hat{B}=\left\{\frac{2(r_{a}+r_{b})}{\left[R_{H}+r_{D}+r_{3}+2(r_{a}+r_{b})\right]}\right\}$$ (A-1b)

$$\hat{C}=\left\{\frac{r_{S}}{\left[R_{H}+r_{S}+r_{4}+2(r_{c}+r_{d})\right]}\right\}$$ (A-1c)

$$\hat{D}=\left\{\frac{2(r_{c}+r_{d})}{\left[R_{H}+r_{S}+r_{4}+2(r_{c}+r_{d})\right]}\right\}$$ (A-1d)

$$\hat{E}=\frac{\left(r_{a}+r_{b}\right)}{\left(r_{a}+r_{b}+r_{c}+r_{d}\right)}$$ (A-1e)

$$\hat{F}=\frac{\left(r_{c}+r_{d}\right)}{\left(r_{a}+r_{b}+r_{c}+r_{d}\right)}.$$ (A-1f)

The double-series ring-array current solutions are then

$$I_{S3}=\left[\frac{\left[1+\hat{A}\hat{E}-(\hat{C}+\hat{D})\hat{F}\right]}{\left[2-\hat{B}\hat{E}-\hat{D}\hat{F}\right]}\right]I_{T}$$ (A-2a)

$$I_{4D}=I_{T}-I_{S3}$$ (A-2b)

$$I_{3}=\hat{A}I_{T}+\hat{B}I_{S3}\approx \frac{r_{D}}{R_{H}}I_{T}$$ (A-2c)

$$I_{4}=\hat{C}I_{T}+\hat{D}I_{4D}\approx \frac{r_{S}}{R_{H}}I_{T}$$ (A-2d)

$$I_{3D}=I_{S3}-I_{3}$$ (A-2e)

$$I_{S4}=I_{4D}-I_{4}$$ (A-2f)

$$I_{S}=I_{T}-I_{4}$$ (A-2g)

$$I_{D}=I_{T}-I_{3}.$$ (A-2h)

The quantized Hall voltage measured between points Y and Z is

$$\begin{array}{l} V_{H}(Y,Z)=R_{H}I_{T}+r_{3}I_{3}+r_{4}I_{4}\\ +2(r_{c}+r_{d})(I_{S3}+I_{S4}), \end{array}$$ (A-3a)

or approximately

$$V_{H}(Y,Z)\approx R_{H}\left[1+\frac{r_{3}r_{D}}{R_{H}R_{H}}+\frac{r_{4}r_{S}}{R_{H}R_{H}}\right]I_{T}.$$ (A-3b)

The exact solutions for the currents and for *V*_H_(Y, Z) in Eq. (A-3a) are slightly different from the results calculated in Sec. 5.1 for the diamond-array with the same device connections. While the double-series ring-array calculation results are not presented in Table 1, they are nearly identical to the double-series diamond-array results when using the same four representative cases of lead and longitudinal resistances. The largest discrepancies are for the case with 10 Ω lead resistances and 1 mΩ longitudinal resistances, where the ring-array current ratio *I*_3_/*I*_T_ is fractionally smaller than the diamond-array current ratio by 1.2 × 10^−10^, and the fractional Hall resistance error [*R*_H_(Y,Z)/*R*_H_] − 1 is 2.4 × 10^−10^ larger. Even though the results are similar for these representative cases, the diamond-array solutions are much simpler to derive and to calculate.

### 11.2 Longitudinal Voltage Configuration

Figure A-2 shows a double-series ring-array connection to the device that could be used for longitudinal voltage measurements if significant antenna noise is present in the sample probe leads. The current solutions are now

$$I_{S3}=\left\{\frac{\left[1+\hat{A}\hat{E}\right]}{\left[2-\hat{B}\hat{E}\right]}\right\}I_{T}$$ (A-4a)

$$I_{3}=\hat{A}I_{T}+\hat{B}I_{S3}\approx \frac{r_{D}}{R_{H}}I_{T}$$ (A-4b)

$$I_{3D}=I_{S3}-I_{3}$$ (A-4c)

$$I_{S4}=I_{T}-I_{S3}=I_{4D}$$ (A-4d)

$$I_{D}=I_{T}-I_{3}.$$ (A-4e)

The double-series ring-array calculation results are not presented in Table 2 for the longitudinal voltage configuration, but the current ratios *I*_3_/*I*_T_ are nearly identical to the double-series diamond-array results when using the same four representative cases of lead and longitudinal resistances. The largest discrepancies are for the case with 10 Ω lead resistances and 1 mΩ longitudinal resistances, where the ring-array current ratio *I*_3_/*I*_T_ is fractionally smaller than the diamond-array current ratio by 6 × 10^−11^.

The longitudinal voltages are

$$V_{x}(2,4)=2r_{b}I_{4D}=2r_{b}I_{S4}$$ (A-5a)

$$V_{x}(2,4)\approx r_{b}\left[1-\frac{r_{D}}{2R_{H}}\right]I_{T}$$ (A-5b)

$$V_{x}(4,6)=2r_{c}I_{S4}$$ (A-5c)

$$V_{x}(4,6)\approx r_{c}\left[1-\frac{r_{D}}{2R_{H}}\right]I_{T}.$$ (A-5d)

Both longitudinal voltages *V_x_*(2,4) and *V_x_*(4,6) have approximate [1 – *r*_D_/2*R*_H_] corrections in the ring-array, whereas there is an approximate [1 – *r*_D_/*R*_H_] correction to *V_x_*(2,4) and no correction to *V_x_*(4,6) in the diamond-array of Sec. 5.2. The largest discrepancies between *V_x_*(2,4) and *V_x_*(4,6) are for the two cases with 10 Ω lead resistances, where the ring-array longitudinal voltages are both fractionally smaller than the diamond-array voltages by 3.9 × 10^−4^. These are small discrepancies because the longitudinal voltages are small.
